# Off-label testosterone therapy is associated with higher long-term cardiovascular risk in men

**DOI:** 10.1016/j.ebiom.2026.106373

**Published:** 2026-07-09

**Authors:** Hatim Kerniss, Philip Curman, Henning Olbrich, Khalaf Kridin, Rohin Francis, David M. Leistner, Uazman Alam, Ralf J. Ludwig

**Affiliations:** a Department of Cardiology, University Heart Centre Frankfurt, University Hospital Frankfurt, Frankfurt/Main, Germany; b Luebeck Institute of Experimental Dermatology, University of Luebeck, Lübeck, Germany; c German Centre for Cardiovascular Research (DZHK), Partner Site Rhine-Main, Frankfurt/Main, Germany; d Dermato-Venereologica Clinic, Karolinska University Hospital, Stockholm, Sweden; e Department of Medical Epidemiology and Biostatistics, Karolinska Institutet, Stockholm, Sweden; f Dermatology and Venereology Division, Department of Medicine (Solna), Karolinska Institutet, Stockholm, Sweden; g Department of Dermatology, University Medical Centre of the State of Schleswig-Holstein, Campus Luebeck, Luebeck, Germany; h Azrieli Faculty of Medicine, Bar-Ilan University, Safed, Israel; i Unit of Dermatology and Skin Research Laboratory, Galilee Medical Centre, Nahariya, Israel; j The Essex Cardiothoracic Centre, Essex, UK; k Institute of Life Course and Medical Sciences, University of Liverpool, Liverpool, UK; l Department of Medicine, University Hospital Aintree, Liverpool University NHS Foundation Trust, Liverpool, UK; m Institute and Comprehensive Centre for Inflammation Medicine, University-Hospital Schleswig-Holstein, Luebeck, Germany

**Keywords:** Testosterone therapy, Safety, Off-label use, Hypogonadism

## Abstract

**Background:**

Testosterone therapy (TT) is increasingly initiated outside classical hypogonadism, but long-term cardiovascular safety in this context remains uncertain. We assessed whether TT initiation without evidence of hypogonadism is associated with higher long-term cardiovascular risk than TT initiation with evidence of hypogonadism.

**Methods:**

In this global retrospective real-world cohort study, men aged 30–75 years initiating TT were identified from secondary, de-identified structured EHR data from 123 healthcare organisations. A prespecified 3-year pre-index washout excluded prior TT and major cardiovascular/thromboembolic events. Men initiating TT without evidence of hypogonadism were compared with those with evidence of hypogonadism using 1:1 propensity-score matching. The primary outcome was major adverse cardiovascular events (MACE); secondary outcomes included separately all-cause mortality, myocardial infarction, ischaemic stroke, cardiac arrest, and heart failure. Acute appendicitis served as a negative-control outcome.

**Findings:**

Among 358,957 TT initiators, 127,152 (35.4%) had no evidence of hypogonadism. After matching, 113,554 pairs were followed for up to 10 years. TT without evidence of hypogonadism was associated with higher risk of MACE (16.53% vs 11.83%; HR 1.51, 1.45–1.56) and all-cause mortality (HR 1.90, 1.79–2.00), with higher risks of ischaemic stroke (HR 1.23, 1.14–1.33), cardiac arrest (HR 1.41, 1.23–1.61), and heart failure (HR 1.32, 1.26–1.39). Race-stratified estimates were directionally consistent but varied in magnitude.

**Interpretation:**

The cardiovascular safety of TT appears context-dependent and less favourable when treatment is initiated without evidence of hypogonadism, with clinically relevant heterogeneity across race/ethnicity, supporting biologically informed prescribing and cardiovascular risk surveillance.

**Funding:**

10.13039/501100001659Deutsche Forschungsgemeinschaft, Schleswig-Holstein Excellence-Chair Program, and Region Stockholm.

**Research in context**

**Evidence before this study**

Previous studies on testosterone therapy and cardiovascular risk have reported mixed results. Earlier observational studies raised concern about increased risks of cardiovascular events, whereas more recent randomised and meta-analytic evidence in men with confirmed hypogonadism has generally not shown a clear excess risk of major cardiovascular events or death under trial conditions. However, much of the existing literature has compared men receiving testosterone therapy with men not receiving it, rather than comparing different groups of testosterone users according to hypogonadism status. In addition, prior work has focused either on selected trial populations, short-term thromboembolic risk, or men with confirmed hypogonadism, leaving uncertainty about long-term cardiovascular safety when testosterone therapy is initiated without evidence of hypogonadism.

**Added value of this study**

This study directly compared new users of testosterone therapy with and without evidence of hypogonadism. By using a new-user design, a 3-year washout period, incident outcome analyses, propensity-score matching, sensitivity analyses, and a negative-control outcome, we sought to reduce bias and strengthen causal inference. We found that about one in three men initiating testosterone therapy had no evidence of hypogonadism. Compared with matched men treated in the setting of hypogonadism, these men had higher long-term risks of major cardiovascular events, death, ischaemic stroke, cardiac arrest, and heart failure. We also observed clinically relevant variation in effect size across racial and ethnic groups.

**Implications of all the available evidence**

Taken together, the available evidence suggests that the cardiovascular safety of testosterone therapy may depend on the clinical and biological context in which treatment is initiated. Trial evidence is most reassuring for carefully selected men with confirmed hypogonadism under structured monitoring, whereas our findings suggest that outcomes may be less favourable when therapy is initiated without evidence of hypogonadism in routine practice. These results support careful diagnostic evaluation before treatment, close cardiovascular risk assessment during follow-up, and further research into mechanisms that may explain differential risk across patient groups.

## Introduction

Testosterone deficiency is common in ageing men and often presents with sexual symptoms, fatigue, and changes in body composition.^1^ Beyond these clinical features, low circulating testosterone has repeatedly been linked to an adverse cardiometabolic profile and higher long-term mortality in observational cohorts, leaving an unresolved question of whether testosterone is simply a marker of underlying health, or a modifiable risk factor for vascular health.^2^ Testosterone therapy (TT) can improve selected symptoms and physiological parameters in men with confirmed hypogonadism, and current guidelines therefore recommend TT primarily for men with consistently low testosterone levels.^3^^,^^4^

However, TT remains one of the most debated therapies in contemporary men's health. On one side, physiological replacement could plausibly improve metabolic health and thereby reduce future cardiovascular risk. On the other, TT may increase risk through mechanisms such as erythrocytosis and alterations in blood-pressure, particularly in middle-aged and older men who carry substantial cardiometabolic comorbidity. This biological tension is mirrored by a conflicted literature, with studies reporting neutral, beneficial, or even potentially harmful cardiovascular effects depending on study design, patient selection, and exposure definitions.^3^, ^4^, ^5^, ^6^, ^7^, ^8^, ^9^ Despite the clear “on-label” and guideline-concordant framework, real-world TT use has expanded markedly over the past two decades, and prescribing patterns increasingly extend beyond classical organic hypogonadism.^5^^,^^6^ Importantly, much of this growth occurred without major new indications, raising concern that a meaningful proportion of TT initiations may occur when biochemical hypogonadism is absent.^7^

Randomised evidence has advanced substantially in the past few years. The TRAVERSE trial, a large cardiovascular outcomes trial in men with hypogonadism and elevated baseline cardiovascular risk, reported non-inferiority of TT vs placebo for major adverse cardiovascular events (MACE) in a closely monitored trial setting.^8^ In parallel, systematic reviews and meta-analyses of randomised trials, including individual-patient and aggregate-data approaches, have generally not shown an excess of cardiovascular events or all-cause mortality with TT in the trial populations studied.^9^ However, trial contexts are necessarily selective: eligibility typically requires evidence of hypogonadism and structured monitoring, and trials cannot fully capture common real-world patterns such as treatment initiated in the absence of guideline-based indication. Importantly, this evidence gap is difficult to address in a randomised trial for ethical reasons. Randomising men without evidence of biochemical deficiency to long-term testosterone exposure would therefore be ethically challenging and unlikely to be broadly implementable at the scale and duration required for hard cardiovascular outcomes. A population-scale, longitudinal, multi-institutional clinical dataset, therefore, provides a pragmatic approach to evaluate long-term cardiovascular safety in this real-world prescribing setting.

As a result, a key question remains open for both practice and public health: among men who receive TT in routine care, does cardiovascular risk differ according to hypogonadism status?

A major translational gap persists because much of the observational literature compares TT users with non-users without reliably separating men with hypogonadism from those treated without clear biochemical evidence.^10^^,^^11^ As a result, it remains unclear whether testosterone-associated cardiovascular safety reflects the drug itself, the biological context in which it is prescribed, or both. Moreover, long-term follow-up and the role of ethnicity as a potential modifier of TT-associated cardiovascular risk remain insufficiently explored in large, treated cohorts. Using a large global, multi-institutional cohort of new testosterone users, we compared men treated with vs without evidence of hypogonadism and assessed long-term incident major adverse cardiovascular events, mortality, and related endpoints over up to 10 years. We further examined heterogeneity across race/ethnicity and baseline haematocrit to explore clinically relevant modifiers of risk.

## Methods

### Study design and data source

**Fig. 1:**
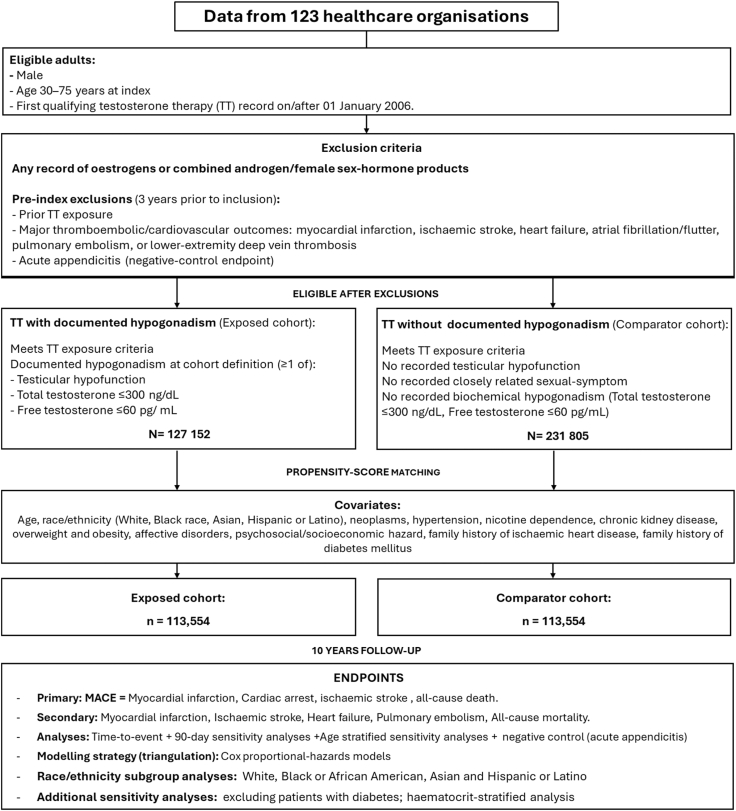
**Study design**. Schematic overview of the retrospective new-user, active-comparator cohort design using secondary, de-identified structured electronic health-record data from the TriNetX Global Collaborative Network. Outcomes were ascertained from day 1 after index until first occurrence of the outcome, last documented clinical activity, or 3650 days after index, whichever occurred first. The primary analysis used 1:1 propensity-score matching, with prespecified sensitivity and subgroup analyses as shown. Abbreviations: PSM, propensity-score matching; TT, testosterone therapy.

We conducted a global retrospective cohort study using routinely collected, de-identified structured EHR data from the TriNetX Global Collaborative Network, a federated real-world data platform operated by TriNetX, LLC. The underlying clinical data are contributed by participating healthcare organisations and include structured diagnoses, procedures, medication records, laboratory values, and encounter information. The main analysis included 123 health care organisations (HCOs) from across North America, South America, Europe, Middle East, Africa and Asia–Pacific. Depending on the network, these may include academic medical centres, non-academic hospital systems, affiliated outpatient clinics, speciality practices, and integrated delivery networks. Analyses followed an established workflow for large-scale federated electronic health-record research on this platform and comprised: (i) cohort identification and index assignment; (ii) pre-index exclusions to minimise reverse causation and incident–prevalent mixing; (iii) covariate ascertainment and propensity-score estimation; (iv) 1:1 propensity-score matching (PSM) with balance assessment; (v) prespecified subgroup and sensitivity analyses; and (vi) multivariable Cox proportional-hazards models in the full cohort. These complementary analytic frameworks were used as part of a methodological triangulation strategy to test robustness across different modelling assumptions. The investigators had no role in treatment initiation, clinical decision-making, or primary data capture. The overall design and cohort definitions are summarised in Fig. 1.

### Ethics statement

The study used secondary, routinely collected structured EHR data from the TriNetX Global Collaborative Network. Analyses were executed within the secure TriNetX analytics environment using de-identified patient-level longitudinal records, including diagnoses, procedures, medication records, laboratory values, and follow-up information. In accordance with privacy-preserving data governance, exported results were restricted to aggregate, de-identified analytical outputs. TriNetX provides de-identified data under the HIPAA Privacy Rule; analyses are performed on aggregated outputs within the platform.^12^ Consequently, this study did not require approval from an institutional review board.

### Study population and definition of eligible patients

Eligible patients were male and aged 30–75 years at the index date, with cohort entry restricted to index events occurring on or after 01 January 2006. The index event was defined as the first qualifying TT record meeting cohort-specific criteria. Detailed cohort identification, eligibility criteria, and index assignment are provided in Supplementary Table S1. We restricted the analytic population to men aged 30–75 years to focus on routine adult testosterone therapy prescribing and to reduce inclusion of younger patients in whom testosterone is more often used in distinct clinical contexts (e.g., pubertal induction), where indications, treatment goals, and risk profiles differ. To align the primary analysis with the age range predominantly studied in major randomised testosterone trials–conducted largely in middle-aged and older men–we prespecified an adult cohort aged 30–75 years.^1^, ^2^, ^3^, ^4^^,^^9^, ^10^, ^11^

Exposure definition: TT exposure was defined by the presence of testosterone medication. To reduce exposure misclassification, patients were excluded if they had oestrogens or combined androgen/female sex-hormone products recorded at cohort definition. TT without evidence of hypogonadism cohort was defined by TT exposure criteria without evidence of hypogonadism or closely related sexual-symptom coding at cohort definition, operationalised as exclusion of testicular hypofunction, decreased libido, sexual dysfunction not due to a substance or known physiological condition, male erectile dysfunction, and biochemical hypogonadism defined by total testosterone ≤300 ng/dL or free testosterone ≤60 pg/mL in the available structured EHR data. Operational definitions used to classify TT users by documentation of hypogonadism are summarised in Supplementary Table S2. TT with hypogonadism cohort: Patients met TT exposure criteria and had hypogonadism defined by ≥1 of testicular hypofunction, total testosterone ≤300 ng/dL, or free testosterone ≤60 pg/mL.

Pre-index exclusions: To approximate a new-user, active-comparator design, individuals with testosterone therapy documentation during the 3-year pre-index washout period were excluded. The same prespecified washout window was used to reduce reverse causation by excluding patients with major cardiovascular or thromboembolic outcomes before treatment initiation, including myocardial infarction, ischaemic stroke, heart failure, atrial fibrillation/flutter, pulmonary embolism, and lower-extremity deep venous thrombosis. Consistent with the incident-outcome approach used for the cardiovascular endpoints, individuals with documented appendicitis during the pre-index washout period were excluded from the risk set for the negative-control analysis. The full set of prespecified washout criteria and baseline exclusions is provided in Supplementary Table S3.

Participants were identified from routinely collected clinical data and were not recruited specifically for this study. Both groups were testosterone therapy initiators; thus, the comparison was not between treated and untreated men, nor between patients and healthy individuals, but between testosterone therapy initiation with vs without evidence of hypogonadism based on available structured EHR data before treatment initiation.

Exposure classification, extended baseline characteristics, and key pre-treatment measurement availability are provided in Supplementary Tables S10, S23, and S24.

### Covariates

To mitigate potential confounding, PSM was performed based on clinically relevant variables after an extensive literature review, including demographics and key comorbidities. We performed 1:1 greedy nearest-neighbour matching with a calliper of 0.1 SD of the logit PSM. The covariates incorporated into the regression model, as well as the baseline characteristics before and after matching, are detailed in Table 1.

### Outcomes

Outcomes were ascertained from day 1 after the index date and continued until the first occurrence of the outcome of interest, the last documented clinical activity within the data source, or 3650 days after index, whichever occurred first. Thus, 10 years represented the maximum follow-up duration. Patients were right-censored at the last documented clinical activity or end of available follow-up. In the matched main cohort, median observed follow-up was 697 days in men initiating TT without evidence of hypogonadism and 1448 days in men initiating TT with evidence of hypogonadism. To evaluate whether differences in observed follow-up duration influenced the findings, we performed an additional sensitivity analysis restricting follow-up to a fixed 2-year window after the index date, using the same matched cohort framework and censoring rules as the main analysis. In the 2-year restricted follow-up sensitivity analysis, median follow-up was more comparable between groups: 695 days in men initiating TT without evidence of hypogonadism and 730 days in men initiating TT with evidence of hypogonadism. The primary outcome was major adverse cardiovascular events (MACE), defined as the composite of myocardial infarction (ICD-10-CM:I21-I23), ischaemic stroke (I63), cardiac arrest (I46), or all-cause death. Prespecified secondary endpoints included: (i) myocardial infarction (I21-I23), (ii) ischaemic stroke (I63), (iii) heart failure overall (I50), (iv) pulmonary embolism (I26), and (v) all-cause mortality. All-cause mortality was defined as recorded deceased status in structured EHR/demographic data or the ICD-10-CM code R99 for ill-defined and unknown cause of mortality. Acute appendicitis was prespecified exclusively as a negative-control endpoint. It was chosen because it is an acute clinical event without a plausible biological relationship to testosterone therapy or cardiovascular risk. For survival analyses, we focused on incident outcome risk by excluding patients with the respective outcome recorded prior to the start of the time-at-risk window (Supplementary Tables S2 and S3).

### Statistical analysis

We performed 1:1 propensity-score matching (PSM) using the TriNetX platform, matching on all prespecified characteristics. Covariate balance was assessed using standardised mean differences (SMD), with summary tables generated before and after matching. Time-to-event outcomes were evaluated using Kaplan–Meier methods with log-rank tests and hazard ratios (HRs) with 95% confidence intervals (CIs) estimated using Cox proportional-hazards models in the matched cohorts. The main estimates were derived from the propensity-score-matched analysis, with adjusted Cox models used as complementary analyses. To quantify adjusted associations in the full cohort, as part of a prespecified complementary analytic approached alongside PSM, we additionally fitted multivariable Cox proportional-hazards models for time-to-event outcomes adjusted for the same prespecified covariates used for PSM (Supplementary Table S9). To reduce protopathic bias and diagnostic intensity immediately after treatment initiation, we performed a sensitivity analysis with a 90-day washout, starting follow-up on day 90 post–index and otherwise retaining identical outcome definitions, censoring rules, and modelling strategy (Supplementary Table S4). To address potential misclassification from incomplete testosterone measurement data and confounding by indication, we performed a laboratory-anchored sensitivity analysis restricted to patients with documented testosterone measurements. The group without evidence of hypogonadism was limited to men with testosterone concentrations above prespecified biochemical thresholds and no hypogonadism diagnosis, sexual-symptom proxy diagnosis, or prior low testosterone measurement before treatment initiation. The comparator group was limited to men with at least two biochemically documented low testosterone measurements, independent of diagnostic coding. Cox regression models were adjusted using the same covariate framework as the main analysis (Supplementary Table S11).

Prespecified subgroup analyses were performed by race/ethnicity (White, Black or African American, Asian, and Hispanic or Latino) and by age, with an additional sensitivity analysis excluding patients with diabetes mellitus at baseline (Supplementary Tables S6–S8). For all stratum-specific analyses, we re-estimated propensity scores and repeated 1:1 propensity-score matching and subsequent time-to-event analyses within each stratum where sample size and event counts allowed stable estimation. Balance diagnostics for these stratum-specific propensity-score matches are provided in Supplementary Tables S12–S18. As an exploratory sensitivity analysis, we repeated adjusted Cox models for the primary outcome within region-restricted cohorts from the United States, Latin America, and Asia–Pacific to assess whether the main findings were directionally consistent across major geographic settings.

Given the propensity of exogenous testosterone to increase haematocrit and the clinical concern that higher haematocrit may amplify thrombotic and vascular risk, we also performed a baseline haematocrit (Hct) sensitivity analysis restricted to men receiving testosterone therapy without evidence of hypogonadism. Patients were stratified by baseline Hct (41–50% vs >50%) and compared using 1:1 propensity-score matching. Index definition, outcome ascertainment, follow-up, and censoring rules were identical to the primary analysis. Balance diagnostics for these stratum-specific propensity-score matches are provided in Supplementary Table S19.

No multiple imputation was performed. The primary analysis did not use complete-case exclusion. The primary endpoint (MACE) was tested two-sided at α = 0.05. Secondary endpoints were assessed with Bonferroni correction across the prespecified endpoint family (myocardial infarction, ischaemic stroke, heart failure, pulmonary embolism, and all-cause mortality), corresponding to α_adj = 0.01 (0.05/5). Accordingly, statistical significance was defined as p < 0.05 for the primary endpoint and p < 0.01 for secondary endpoints (two-sided).

### Role of funders

The funders had no role in study design, data collection, data analysis, data interpretation, writing of the report, or the decision to submit the manuscript for publication. The corresponding author had final responsibility for the decision to submit for publication.

## Results

### Testosterone therapy use patterns and long-term cardiovascular outcomes

Before propensity-score matching, a total of 358,957 men aged 30–75 years receiving TT were identified, including approximately one third (n = 127,152) receiving TT without evidence of hypogonadism and 231,805 receiving TT with hypogonadism. Men with hypogonadism carried a markedly higher cardiometabolic comorbidity burden at baseline, including obesity (23.2% vs 6.5%, p < 0.001), hypertension (39.1% vs 14.0%, p < 0.001), chronic kidney disease (4.2% vs 1.6, p < 0.001) and nicotine dependence (10% vs 4.2%, p < 0.001). After 1:1 propensity-score matching, 113,554 well-balanced pairs remained, with substantially improved covariate balance vs the pre-match cohorts (Table 1).

**Table 1:** Baseline characteristics before and after propensity-score matching.

Characteristic	Before PSM			After PSM		
	TT without documented hypogonadism N = 127,152	TT with documented hypogonadism N = 231,805	SMD	TT without documented hypogonadism N = 113,554	TT with documented hypogonadism N = 113,554	SMD
**Age at index, years,** (mean ± SD)	51.0 ± 11.4	50.5 ± 11.1	0.045	50.2 ± 11.4	50.1 ± 11.2	0.014
**White,** n (%)	94,682 (74.5%)	185,418 (80.0%)	0.132	89,555 (78.9%)	89,033 (78.4%)	0.011
**Hispanic or Latino,** n (%)	6077 (4.8%)	12,764 (5.5%)	0.033	5614 (4.9%)	5761 (5.1%)	0.006
**Black or African American,** n (%)	6943 (5.5%)	17,472 (7.5%)	0.084	6911 (6.1%)	6776 (6.0%)	0.005
**Asian,** n (%)	1834 (1.4%)	4310 (1.9%)	0.033	1828 (1.6%)	1772 (1.6%)	0.004
**Chronic kidney disease,** n (%)	1992 (1.6%)	9747 (4.2%)	0.158	1970 (1.7%)	2014 (1.8%)	0.003
**Socioeconomic/psychosocial hazards,** n (%)	641 (0.5%)	4123 (1.8%)	0.120	638 (0.6%)	617 (0.5%)	0.002
**Family history of ischaemic heart disease,** n (%)	1463 (1.2%)	8419 (3.6%)	0.163	1457 (1.3%)	1412 (1.2%)	0.004
**Overweight and obesity,** n (%)	8258 (6.5%)	53,752 (23.2%)	0.483	8258 (7.3%)	8387 (7.4%)	0.004
**Essential hypertension,** n (%)	17,855 (14.0%)	90,531 (39.1%)	0.591	17,855 (15.7%)	17,842 (15.7%)	<0.001
**Affective disorders,** n (%)	8474 (6.7%)	44,840 (19.3%)	0.384	8469 (7.5%)	8601 (7.6%)	0.004
**Nicotine dependence** n (%)	5346 (4.2%)	23,284 (10.0%)	0.229	5316 (4.7%)	5401 (4.8%)	0.004
**Family history of diabetes mellitus,** n (%)	569 (0.4%)	4211 (1.8%)	0.130	568 (0.5%)	554 (0.5%)	0.002
**Neoplasms,** n (%)	12,628 (9.9%)	56,962 (24.6%)	0.395	12,627 (11.1%)	12,707 (11.2%)	0.002

Baseline characteristics are shown before and after 1:1 propensity-score matching comparing TT with documented hypogonadism vs TT without documented hypogonadism. Matching used greedy nearest-neighbour matching on the propensity score with a calliper of 0.1 SD of the logit. Covariate balance was assessed using standardised mean differences (SMD), with absolute SMD values closer to 0 indicating better balance. p values are descriptive (two-sided) and may be statistically significant even with minimal imbalance due to large sample size; SMD is emphasised for balance assessment.

Abbreviations: PSM, propensity-score matching; SMD, standardised mean difference; SD, standard deviation; TT, testosterone therapy; CKD, chronic kidney disease.

**Fig. 2:**
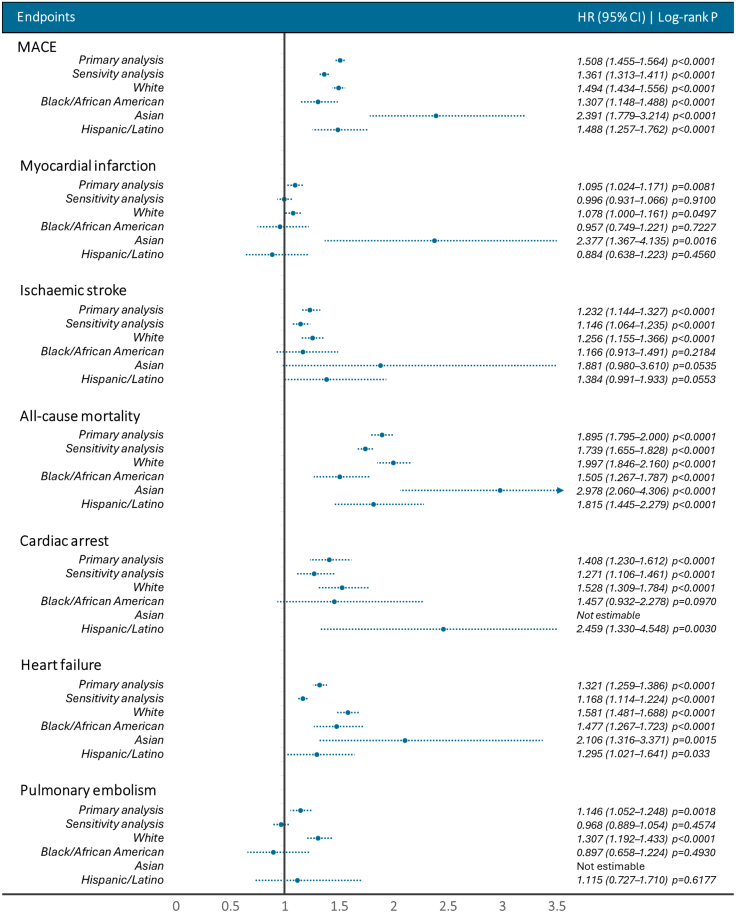
**Long-term cardiovascular outcomes with testosterone therapy in men with vs without evidence hypogonadism**. Forest plot showing hazard ratios (HRs) with 95% confidence intervals (CIs) for prespecified outcomes. The primary comparison contrasts TT without evidence of documented hypogonadism (off-label pattern in routine care) vs TT with hypogonadism. The overall main analysis was performed in 1:1 propensity-score–matched cohorts (age 30–75 years) with follow-up up to 10 years. A prespecified 90-day sensitivity analysis excluded early events by starting time-at-risk on day 90 after the index date. Race/ethnicity-stratified analyses repeated matching within each stratum (White, Black or African American, Asian, Hispanic or Latino). Log-rank p-values derive from Kaplan–Meier comparisons. The vertical reference line indicates HR = 1.0 (no association). Arrows indicate CIs extending beyond the plotted range. Em dashes denote estimates not shown because event counts were insufficient for stable estimation as prespecified. **Abbreviations**: CI, confidence interval; HR, hazard ratio; MACE, major adverse cardiovascular events; TT, testosterone therapy.

**Fig. 3:**
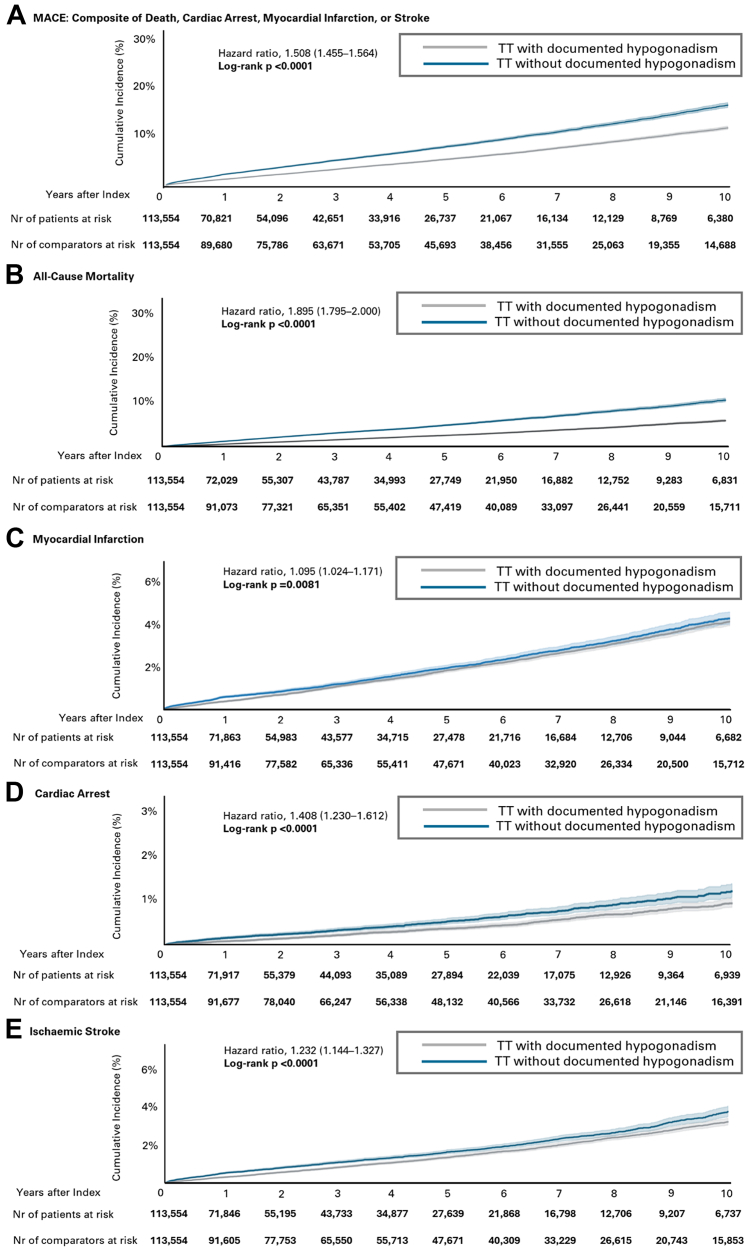
**Cumulative incidence of major cardiovascular outcomes after testosterone therapy in men with vs without hypogonadism**. Kaplan–Meier curves show cumulative incidence (%) from the index date to 10 years in 1:1 propensity-score–matched men receiving testosterone therapy with documented hypogonadism vs testosterone therapy without documented hypogonadism. Panels display: (A) major adverse cardiovascular events (MACE), (B) all-cause mortality, (C) myocardial infarction, (D) cardiac arrest, and (E) ischaemic stroke. Shaded bands indicate 95% confidence intervals. Hazard ratios (HRs) with 95% CIs are from Cox proportional-hazards models in the matched cohorts; log-rank p values are shown for between-group comparisons. MACE was defined as a composite of all-cause death, cardiac arrest, myocardial infarction, or ischaemic stroke. **Abbreviations**: CI, confidence interval; HR, hazard ratio; MACE, major adverse cardiovascular events; TT, testosterone therapy.

In the matched population, TT without evidence of hypogonadism was associated with higher long-term cardiovascular risk across most prespecified endpoints (Figs. 2 and 3). The 10-year absolute risk of MACE was 16.53% compared with 11.83% in men with hypogonadism, corresponding to an HR of 1.508 (95% CI 1.455–1.564; Log-rank p < 0.001). This association persisted in the prespecified 90-day sensitivity analysis (HR 1.361, 95% CI 1.313–1.411; Log-rank p < 0.001). All-cause mortality showed a similarly pronounced elevation (10.71% vs 5.99%; HR 1.895, 95% CI 1.795–2.000; Log-rank p < 0.001), again with confirmation in the sensitivity analysis (HR 1.739, 95% CI 1.655–1.828; Log-rank p < 0.001). Ischaemic stroke demonstrated a 10-year increase (3.84% vs 3.29%; HR 1.232, 95% CI 1.144–1.327; Log-rank p < 0.001), and this persisted in sensitivity analysis (HR 1.146, 95% CI 1.064–1.235; Log-rank p < 0.001). Myocardial infarction showed a marginal but statistically significant increase at 10 years (4.32% vs 4.16%; HR 1.095, 95% CI 1.024–1.171; Log-rank p = 0.008), although no association was observed in the 90-day sensitivity analysis (HR 0.996, 95% CI 0.931–1.066; Log-rank p = 0.910). Cardiac arrest risk was also elevated (1.23% vs 0.95%; HR 1.408, 95% CI 1.230–1.612; Log-rank p < 0.001) with consistent findings in sensitivity analysis (HR 1.271, 95% CI 1.106–1.461; p < 0.001).

Heart-failure outcome was consistently higher in men receiving TT without evidence of hypogonadism and occurred in 8.60% vs 7.23% (HR 1.321, 95% CI 1.259–1.386; Log-rank p < 0.001) with persistence in the sensitivity analysis (HR 1.168, 95% CI 1.114–1.224; Log-rank p < 0.001). Pulmonary embolism showed a statistically significant 10-year increase (2.50% vs 2.43%; HR 1.146, 95% CI 1.052–1.248; Log-rank p = 0.002), although no association was observed in the sensitivity analysis (HR 0.968, 95% CI 0.889–1.054; p = 0.457). The prespecified negative-control endpoint, acute appendicitis, showed no association at 10 years (HR 0.877, 95% CI 0.744–1.035; Log-rank p = 0.119) and remained null in sensitivity analyses (HR 0.884, 95% CI 0.746–1.048; Log-rank p = 0.156), supporting specificity of the cardiovascular findings. In the 2-year restricted follow-up sensitivity analysis, the associations remained significant and was numerically stronger than in the main analysis (Supplementary Table S5). Overall, these results were directionally concordant with fully adjusted Cox proportional-hazards models in the full cohorts (Supplementary Table S9).

In the laboratory-anchored sensitivity analysis, testosterone therapy initiation without evidence of hypogonadism, remained associated with higher MACE risk compared with men with at least two documented low testosterone measurements before treatment (HR 1.52, 95% CI 1.35–1.71; Log-rank p < 0.0001). Associations were directionally consistent for secondary outcomes, with statistical significance for myocardial infarction, all-cause mortality, heart failure, and ischaemic stroke (Supplementary Table S11). In the sensitivity analysis excluding men with diabetes mellitus, the excess risk associated with TT without evidence of hypogonadism persisted, including MACE (HR 1.765, 95% CI 1.686–1.847; p < 0.001), myocardial infarction (HR 1.594, 95% CI 1.459–1.740; Log-rank p < 0.001), ischaemic stroke (HR 1.607, 95% CI 1.464–1.765; p < 0.001), and all-cause mortality (HR 1.953, 95% CI 1.840–2.073; Log-rank p < 0.001) (Supplementary Table S20). In a separate analysis restricted to men receiving testosterone therapy without evidence of hypogonadism, baseline haematocrit >50% (vs 41–50%) was associated with a modestly higher risk of MACE (HR 1.120, 1.002–1.251; Log-rank p = 0.046) and myocardial infarction (HR 1.267, 1.046–1.536; Log-rank p = 0.015) (Supplementary Table S21).

### Race/ethnicity subgroup analyses reveal marked variation in cardiovascular risk

Before propensity-score matching, off-label TT use was likewise frequent across race/ethnicity strata, comprising 94,682 of 280,100 White men (33.8%), 6943 of 24,415 Black/African American men (28.4%), 1834 of 6144 Asian men (29.9%), and 6077 of 18,841 Hispanic/Latino men (32.3%) receiving TT without evidence of hypogonadism. After 1:1 propensity-score matching within each race/ethnicity stratum, well-balanced pairs were retained with excellent covariate balance relative to the pre-match cohorts (all absolute SMDs <0.05). Race/ethnicity subgroup analyses demonstrated marked heterogeneity in the magnitude of cardiovascular associations, although the directional pattern remained largely consistent (Figs. 2 and 3). The strongest MACE and mortality associations were observed in Asian men (MACE HR 2.391; mortality HR 2.978), followed by White (MACE HR 1.494; mortality HR 1.883), Hispanic/Latino (MACE HR 1.488; mortality HR 1.815), and Black/African American men MACE HR 1.307; mortality HR 1.505, all Log-rank p < 0.0001). Heterogeneity was most pronounced for myocardial infarction: the association was borderline in White men (HR 1.078, Log-rank p = 0.0497), absent in Black/African American (HR 0.957, Log-rank p = 0.7227) and Hispanic/Latino men (HR 0.88, Log-rank p = 0.456), yet markedly increased in Asian men (HR 2.377, Log-rank p = 0.0016). Ischaemic stroke was significantly increased in White men (HR 1.256, Log-rank p < 0.0001) but did not reach significance in other groups, although an increased trend in Asian (HR 1.881, Log-rank p = 0.0535) and Hispanic/Latino men (HR 1.384, Log-rank p = 0.0553) is observed. Heart-failure outcomes were elevated in most racial and ethnic groups, particularly in Black (HR 1.477, Log-rank p < 0.0001), White (HR 1.289, Log-rank p < 0.0001) and Asian men (HR 2.106, Log-rank p = 0.0015). The negative-control endpoint was null across all racial/ethnic subgroups, reinforcing the specificity of cardiovascular risk associations (Supplementary Table S7). In exploratory heterogeneity testing across race-specific matched estimates, evidence of heterogeneity was observed for MACE, myocardial infarction, all-cause mortality, and heart failure, whereas no statistically significant heterogeneity was observed for ischaemic stroke (Supplementary Table S22). Because several Asian subgroup analyses were based on limited event numbers, these findings should be interpreted as exploratory. In exploratory region-restricted sensitivity analyses, the association between testosterone therapy initiation without evidence of hypogonadism and MACE remained directionally consistent across the United States (HR 1.37, 95% CI 1.32–1.43; Log-rank p < 0.0001), Latin America (HR 1.87, 95% CI 1.22–2.86; Log-rank p = 0.0041), and Asia–Pacific cohorts (HR 3.57, 95% CI 2.88–4.43; Log-rank p < 0.0001). This pattern was directionally aligned with the race/ethnicity-stratified analysis in the Global Network.

## Discussion

In this large real-world cohort of more than 350,000 men treated with testosterone, we found three overarching signals. First, seemingly off-label TT use without diagnosis of hypogonadism was remarkably frequent, accounting for roughly one third of all TT prescriptions. Second, compared with men treated in the setting of hypogonadism, those treated without such evidence had consistently higher long-term risks of major adverse cardiovascular and cerebrovascular events, heart failure, cardiac arrest, ischaemic stroke, and all-cause mortality. Third, race/ethnicity-stratified analyses revealed substantial risk differences. These findings are translationally relevant because they suggest that testosterone-associated cardiovascular risk may not be uniform across treated populations, but instead may depend on the biologic and diagnostic context in which therapy is initiated. This interpretation is supported by the observed persistence of stroke, cardiac arrest, and heart-failure signals, together with the higher risk seen among men with elevated baseline haematocrit. Taken together, these data raise the possibility that mechanisms such as erythrocytosis-related hyperviscosity, haemodynamic effects, or context-specific susceptibility may contribute to differential cardiovascular safety.^3^^,^^4^

Much of the public and regulatory debate over the past decade has centred on whether TT increases cardiovascular risk overall.^10^^,^^13^^,^^14^ Early observational studies suggested higher rates cardiovascular risk among TT users, whereas later cohorts and meta-analyses have reported neutral or even lower cardiovascular risk in appropriately selected hypogonadal men.^8^^,^^9^^,^^11^^,^^15^^,^^16^ For instance, in a recent trial in Kleinfelter's syndrome, TT was associated with lower all-cause mortality (adjusted hazard ratio (95% CI); 0.56 (0.37–0.85), whilst Incidence of MACE was comparable between TT and non-TT cohort.^17^ These inconsistencies have fuelled considerable controversy and were one of the reasons why the U.S. Food and Drug Administration (FDA) issued safety communications in the mid-2010s, warning about possible cardiovascular harm and clarifying that TT was not approved for age-related testosterone decline alone.^18^, ^19^, ^20^ Our results extend and nuance the existing evidence base in several ways. Current guidelines restrict testosterone therapy to men with biochemically confirmed hypogonadism and explicitly discourage its use in eugonadal men.^3^^,^^4^^,^^21^ However, real-world prescribing frequently occurs without guideline-concordant diagnostic testing, and a substantial proportion of treated men have pre-treatment testosterone values within the reference range.^19^^,^^20^ Importantly, pivotal cardiovascular safety evidence primarily pertains to men with confirmed hypogonadism, leaving the safety profile of off-label use insufficiently characterised.^8^^,^^9^^,^^16^ In our study, roughly one in three men receiving TT had no recorded diagnosis of hypogonadism, underscoring substantial off-label use at scale. This aligns with prior reports showing that TT prescribing has increasingly expanded beyond classical organic hypogonadism.^5^^,^^8^^,^^9^^,^^16^^,^^19^^,^^20^^,^^22^ Notably, much of this rise occurred without the introduction of major new approved indications, fuelling concerns that a sizeable share of TT is initiated in men without clear biochemical hypogonadism.^5^^,^^22^

By directly comparing on-label TT (evidence of hypogonadism) vs off-label TT (no evidence of hypogonadism) in a large propensity-matched cohort, our study addresses this clinically and guideline-relevant evidence gap. In matched analyses, TT without evidence of hypogonadism was associated with higher long-term hazards for MACE, mortality, stroke, cardiac arrest, and heart-failure outcomes, with confirmation for several endpoints in a prespecified sensitivity analysis, subgroups and directional concordance in fully adjusted Cox models.

The pattern across endpoints is also informative. In our main analysis, myocardial infarction showed a modest 10-year excess risk but became neutral in the sensitivity analysis, whereas stroke, cardiac arrest, and heart-failure signals persisted. This may suggest that early risk dynamics and outcome ascertainment differ by endpoint, and that embolic/arrhythmic or haemodynamic pathways (e.g., blood pressure elevation, erythrocytosis-related viscosity, or fluid retention) could be more relevant than atherosclerotic plaque-driven myocardial infarction in certain settings.^3^^,^^9^^,^^23^ The null negative-controls endpoint across analyses further support that the observed associations are not simply reflecting generalised outcome inflation. Subgroup analyses by age and race/ethnicity provide additional granularity but need careful interpretation. In age-stratified sensitivity analyses, the relative excess risk for MACE was relatively similar in younger and older men, yet the pattern across endpoints differed. Younger men showed proportionally stronger associations for ischaemic stroke and cardiac arrest, whereas the excess risk of all-cause mortality was more pronounced in older men. Clinically, this argues against a simplistic paradigm in which TT is considered “safe” in younger men and “risky” only in older, frailer patients. At the same time, testosterone therapy initiation without documented evidence of hypogonadism should not be interpreted as confirmed eugonadism. This distinction is clinically important because non-medical or performance-related androgen use may involve supraphysiological dosing, cycling, formulation switching, and intermittent use, patterns that differ substantially from monitored physiological replacement therapy. Prior studies have linked supraphysiological anabolic-androgenic steroid exposure to adverse cardiovascular phenotypes, including dyslipidaemia, blood pressure elevation, erythrocytosis, myocardial dysfunction, and accelerated coronary atherosclerosis. Therefore, if high-dose or performance-related testosterone use was more common among men initiating therapy without documented evidence of hypogonadism, this may represent one plausible explanation for the observed excess risk.^24^

Race/ethnicity stratification revealed even more pronounced differences. While MACE and overall mortality were consistently increased across all strata, the magnitude of risk was highest in Asian men, followed by White and Hispanic/Latino men, with somewhat smaller relative elevations in Black/African American men. The marked hazard ratios for MACE, mortality, and myocardial infarction in Asian men should be interpreted cautiously because sample sizes were smaller and confidence intervals wider than in White cohorts. Nevertheless, they raise the possibility that underlying ethnic differences in cardiovascular risk profiles, body composition, pharmacokinetics, or susceptibility to androgen-related changes in haemostasis and blood pressure could modulate the impact of TT.^25^, ^26^, ^27^ Data on TT safety in Asian populations remain sparse, and existing studies are generally underpowered to robustly address cardiovascular endpoints. Further work in more diverse, non-Western cohorts is therefore needed to confirm or refute our subgroup observations and to explore potential mechanisms.

Important limitations should be acknowledged. As with all observational analyses, residual and unmeasured confounding cannot be excluded. Confounding by indication remains a central concern. Although we used an active-comparator, new-user design among TT initiators, propensity-score matching, complementary adjusted Cox models, and laboratory-anchored sensitivity analyses, these approaches can only reduce, not eliminate, confounding related to the clinical context of TT initiation. Men initiating TT without documented hypogonadism data may have differed from those with evidence of hypogonadism in unmeasured ways, including symptom burden, prescribing setting, clinician decision-making, patient preference, lifestyle factors, non-medical or performance-related use, and monitoring intensity. Accordingly, the findings should be interpreted as associations according to available hypogonadism evidence and documented prescribing context, not as causal estimates of TT itself.

Testosterone prescribing was not standardised and may have varied across countries, healthcare systems, primary and specialist care, and private settings. Although exploratory region-restricted analyses showed directionally consistent findings, regional differences in diagnostic work-up, prescription capture, laboratory availability, and documentation may still have contributed to exposure misclassification and residual confounding.

Treatment duration, adherence, discontinuation, formulation-specific route, formulation switching, dose, and cumulative exposure could not be reliably reconstructed in the available structured EHR data. Therefore, the analysis should be interpreted as an index-exposure, intention-to-treat–like comparison rather than as a strictly on-treatment, formulation-specific, or dose–response analysis. Intermittent use, early discontinuation, or treatment interruptions in either group may have influenced the observed associations, but could not be assessed reliably. Documentation of hypogonadism in routine clinical practice may be incomplete, and ascertainment of hypogonadism status in structured EHR data is inherently imperfect. In contrast, the available structured EHR data did not uniformly capture timing of blood sampling, fasting status, assay characteristics, repeated confirmatory measurements, symptom severity, or clinical reasoning. Therefore, some men classified as receiving TT with evidence of hypogonadism may have been identified on the basis of a single low testosterone value or diagnostic coding alone, whereas some men classified as receiving TT without evidence of hypogonadism based on available data may have had true biochemical deficiency or symptoms that were not captured. Accordingly, absence of hypogonadism evidence in the available structured EHR data should be interpreted as a proxy for potentially non–guideline-concordant TT prescribing, rather than as a definitive measure of off-label use. Because cohort assignment relied on available structured clinical data, the absence of documented hypogonadism, testosterone measurements, hypogonadal symptoms, or prior cardiovascular events should not be equated with their definitive clinical absence. Consequently, some men classified as receiving TT “without evidence of hypogonadism” may have had true biochemical deficiency that was not captured in the available data. To reduce potential misclassification, we added a laboratory-anchored sensitivity analysis restricted to patients with documented testosterone measurements; in this analysis, the primary association remained directionally consistent and statistically significant.

Despite these limitations, this study provides a large-scale, head-to-head comparison of men receiving testosterone therapy with vs without evidence of hypogonadism, with up to 10 years of follow-up, race/ethnicity stratification, rigorous propensity-score matching, and complementary modelling approaches. Importantly, this question is unlikely to be resolved by conventional randomised designs in the near term: a long-term trial that intentionally enrols and randomises men without evidence of biochemical deficiency to testosterone exposure would face substantial ethical constraints, as guideline-based care requires confirmation of hypogonadism before treatment initiation. The central message is clear—approximately one third of men receiving testosterone therapy lacked evidence of hypogonadism, and this prescribing pattern was associated with a clinically meaningful excess risk of major adverse cardiovascular and cerebrovascular events over long-term follow-up. Together with randomised evidence in selected trial populations, our findings emphasise that cardiovascular safety may depend on prescribing context, reinforcing the importance of guideline-based diagnostic work-up before treatment initiation.

## Contributors

HK and RJL take responsibility for the integrity of the work, including the study concept, cohort and variable definitions, analytical strategy, interpretation of the results, and accuracy of the reported analyses. Study concept and design: HK and RJL. Analysis, or interpretation of data: HK, PC, HO, KK, RF, DML, UA, and RJL. Draughting of the manuscript: HK. Critical revision of the manuscript for important intellectual content: HK, PC, HO, KK, RF, DML, UA and RJL. Statistical analysis: HK. Administrative, technical, or material support: HK and PC. Supervision: PC and RJL. All authors read and approved the final version of the manuscript and had final responsibility for the decision to submit for publication.

## Data sharing statement

The data supporting this study are derived from the TriNetX Global Collaborative Network. In accordance with TriNetX data-sharing policies and privacy safeguards, individual-level patient data were not accessible to the authors and cannot be publicly shared. Aggregated analytical outputs used in this study are available from the corresponding author upon reasonable request. Access to the underlying TriNetX data requires a data use agreement with TriNetX.

## Declaration of interests

RJL reports research funding from EUROIMMUN, Dompé pharma, Novartis, and Sanofi. All other authors declare no competing interests.
