# Supplementary material for: Off-label testosterone therapy is associated with higher long-term cardiovascular risk in men
Source: eBioMedicine. 2026 Jul 9;130:106373. doi: 10.1016/j.ebiom.2026.106373 (PMC13380502; doi:10.1016/j.ebiom.2026.106373)
Supplement: Supplementary Material [file mmc1.docx]

**Supplementary Materials**

**Supplementary Table S1. Cohort identification, eligibility criteria, and index assignment**

| ***Domain*** | ***Phenotype / criterion*** | ***Coding system*** | ***Codes*** |
| --- | --- | --- | --- |
| ***Eligibility*** | *Age 30–75 years* | *—* | *—* |
|  | *Male sex* | *HL7V3* | *Gender:M* |
|  | *Exclusion: oestrogens* |  | *4100* |
|  | *Exclusion: androgens female sex hormones in combination* | *ATC* | *G03EK* |
| ***TT ascertainment*** | *Testosterone* | *RxNorm* | *10379* |
|  | *Injection, testosterone undecanoate, 1 mg* | *HCPCS* | *C9023* |
|  | *Injection, testosterone cypionate, 1 mg* | *HCPCS* | *J1071* |
|  | *Injection, testosterone cypionate, up to 100 mg* | *HCPCS* | *J1070* |
|  | *Injection, testosterone propionate, up to 100 mg* | *HCPCS* | *J3150* |
|  | *Injection, testosterone cypionate, 1 cc, 200 mg* | *HCPCS* | *J1080* |
|  | *Injection, testosterone suspension, up to 50 mg* | *HCPCS* | *J3140* |
|  | *Injection, testosterone undecanoate, 1 mg* | *HCPCS* | *J3145* |
|  | *Injection, testosterone undecanoate, 1 mg* | *HCPCS* | *C9023* |
|  | *Injection, testosterone cypionate, 1 mg* | *HCPCS* | *J1071* |
|  | *Injection, testosterone cypionate, up to 100 mg* | *HCPCS* | *J1070* |
|  | *Injection, testosterone propionate, up to 100 mg* | *HCPCS* | *J3150* |
|  | *Injection, testosterone cypionate, 1 cc, 200 mg* | *HCPCS* | *J1080* |
|  | *Injection, testosterone suspension, up to 50 mg* | *HCPCS* | *J3140* |
|  | *Injection, testosterone undecanoate, 1 mg* | *HCPCS* | *J3145* |

*Testosterone therapy ascertainment included RxNorm testosterone medication terminology and procedure-based testosterone administration codes. RxNorm 10379 represents testosterone at the ingredient level and includes testosterone products across multiple dose-form groups, including topical gel, topical solution, transdermal, nasal, oral, buccal, implant, and injectable preparations. Procedure-based codes primarily capture injectable testosterone administration. To reduce exposure misclassification, patients with oestrogens (RxNorm 4100) or combined androgen/female sex-hormone products (ATC G03EK) were not eligible.*

***Abbreviations:*** *ATC, Anatomical Therapeutic Chemical classification; HCPCS, Healthcare Common Procedure Coding System; HL7, Health Level Seven; RxNorm, normalised naming system for drugs; TT, testosterone therapy.*

**Supplementary Table S2. Cohort definitions**

| *Domain* | *Term* | Coding system | *Codes* |
| --- | --- | --- | --- |
| *TT with evidence of hypogonadism*  *must have ≥1* | Testicular hypofunction | UMLS:ICD10CM | E29.1 |
|  | Total testosterone ≤300 ng/dL | TNX:LG; UMLS:LOINC | LG11398-1; 2986-8 |
|  | Free testosterone ≤60 pg/mL | UMLS:LOINC | 2991-8 |
| *TT without evidence of hypogonadism and*  *Symptom proxies*  *cannot have* | *Testicular hypofunction* | UMLS:ICD10CM | E29.1 |
|  | *Total testosterone ≤300 ng/dL* | TNX:LG; UMLS:LOINC | LG11398-1; 2986-8 |
|  | *Free testosterone ≤60 pg/mL* | UMLS:LOINC | 2991-8 |
|  | *Decreased libido* | UMLS:ICD10CM | R68.82 |
|  | *Sexual dysfunction not due to a substance or known physiological condition* | UMLS:ICD10CM | F52 |
|  | *Male erectile dysfunction* | UMLS:ICD10CM | N52 |
| Pre-index exclusions | TT Therapy (all forms) | RxNorm / procedure codes | 10379; C9023; J1070; J1071; J1080; J3140; J3145; J3150 |
|  | Prior venous thromboembolism | UMLS:ICD10CM | I82.4 |
|  | Prior pulmonary embolism | UMLS:ICD10CM | I26 |
|  | Prior atrial fibrillation/flutter | UMLS:ICD10CM | I48 |
|  | Prior heart failure | UMLS:ICD10CM | I50 |
|  | Prior ischaemic stroke | UMLS:ICD10CM | I63 |
|  | Prior acute myocardial infarction | UMLS:ICD10CM | I21–I23 |
|  | *Negative control endpoint (Acute appendicitis)* | UMLS:ICD10CM | K35 |

*TT with evidence of hypogonadism required ≥1 of the following at or before index: testicular hypofunction and/or biochemical hypogonadism (total testosterone ≤300 ng/dL or free testosterone ≤60 pg/mL). TT without evidence of hypogonadism required TT exposure but excluded any recorded diagnostic or laboratory of hypogonadism, as well as symptom proxy codes for sexual dysfunction. Pre-index exclusions were applied as a washout to reduce reverse causation and ensure incident outcome ascertainment during follow-up. Patients with any listed diagnosis recorded within the prespecified washout window prior to the index date were excluded. Acute appendicitis was prespecified as a negative control endpoint to probe for residual confounding and non-specific associations.*

***Abbreviations:*** *TT, testosterone therapy; UMLS, Unified Medical Language System; ICD-10-CM, International Classification of Diseases, 10th Revision, Clinical Modification; LOINC, Logical Observation Identifiers Names and Codes; TNX, TriNetX laboratory code.*

**Supplementary Table S3. cardiovascular/cerebrovascular outcomes**

| Endpoint | Code system | Codes | Notes |
| --- | --- | --- | --- |
| MACE (primary composite) | ICD-10-CM | I21-23, I63, I46, R99, Deceased | Composite: Myocardial infarction, Ischaemic stroke, Cardiac arrest, mortality. |
| Myocardial infarction | ICD-10-CM | I21, I22, I23 | Acute MI, subsequent MI. |
| Ischaemic stroke | ICD-10-CM | I63 | Cerebral infarction. |
| Heart failure | ICD-10-CM | I50 | Heart failure diagnosis. |
| Pulmonary embolism | ICD-10-CM | I26 | Pulmonary embolism |
| Cardiac arrest | ICD-10-CM | I46 | Cardiac arrest |
| All-cause mortality | TriNetX / ICD-10-CM | Deceased flag and/or R99 | Death |
| Negative control | ICD-10-CM | K35 | Acute appendicitis |

*A pre-index washout was implemented such that any occurrence of the above diagnoses within 3 years and 1 day prior to the first qualifying TT record led to exclusion, as configured in the cohort query. Acute appendicitis (K35) was included as a negative-control diagnosis within the washout specification.****Abbreviations****: ICD-10-CM, International Classification of Diseases, 10th Revision, Clinical Modification; TT, testosterone therapy.*

**Supplementary Table S4. Main analysis and 90-day lag sensitivity analysis**

|  | *Main analysis* | | | | *90-day lag sensitivity analysis* | | | |
| --- | --- | --- | --- | --- | --- | --- | --- | --- |
| *Outcome* | ***HR (95% CI)*** | ***Log-rank p*** | ***PH χ²*** | ***PH p*** | ***HR (95% CI)*** | ***Log-rank p*** | ***PH χ²*** | ***PH p*** |
| *MACE* | *1.508 (1.455–1.564)* | *<0.0001* | *20.68* | *<0.001* | *1.361 (1.313–1.411)* | *<0.0001* | *0.06* | *0.801* |
| *Negative control* | *0.877 (0.744–1.035)* | *0.1192* | *1.62* | *0.203* | *0.884 (0.746–1.048)* | *0.1561* | *1.14* | *0.286* |
| *Myocardial infarction* | *1.095 (1.024–1.171)* | *0.0081* | *8.28* | *0.004* | *0.996 (0.931–1.066)* | *0.9100* | *0.45* | *0.501* |
| *Ischaemic stroke* | *1.232 (1.144–1.327)* | *<0.0001* | *7.99* | *0.005* | *1.146 (1.064–1.235)* | *0.0003* | *0.04* | *0.838* |
| *Heart failure* | *1.321 (1.259–1.386)* | *<0.0001* | *51.06* | *<0.001* | *1.168 (1.114–1.224)* | *<0.0001* | *2.58* | *0.108* |
| *Pulmonary embolism* | *1.146 (1.052–1.249)* | *0.0018* | *16.99* | *<0.001* | *0.968 (0.889–1.054)* | *0.4574* | *1.68* | *0.195* |
| *Cardiac arrest* | *1.408 (1.231–1.611)* | *<0.0001* | *4.43* | *0.035* | *1.271 (1.106–1.461)* | *0.0007* | *0.80* | *0.372* |
| *All-cause mortality* | *1.895 (1.805–1.989)* | *<0.0001* | *4.79* | *0.029* | *1.739 (1.655–1.828)* | *<0.0001* | *1.97* | *0.161* |

*Hazard ratios were derived from platform-based Cox proportional-hazards models. Platform-reported proportionality diagnostics are presented as χ² statistics and p values and should be interpreted as qualitative indicators of possible time-varying relative hazards. A p value <0.05 suggests evidence of non-proportional hazards. For endpoints with possible non-proportional hazards, HRs should be interpreted as average relative hazard estimates over the corresponding follow-up period. The 90-day post-index lag sensitivity analysis was performed to assess whether early post-index risk dynamics contributed to this pattern. MACE = major adverse cardiovascular events; PH = proportional hazards; CI = confidence interval.*

**Supplementary Table S5. Two-year restricted follow-up sensitivity analysis**

| **Outcome** | **HR** | **95% CI** | **Log-rank p** |
| --- | --- | --- | --- |
| **MACE** | 1.805 | 1.693–1.924 | <0.0001 |
| **Myocardial infarction** | 1.448 | 1.279–1.639 | <0.0001 |
| **Ischaemic stroke** | 1.405 | 1.233–1.601 | <0.0001 |
| **Cardiac arrest** | 1.874 | 1.458–2.409 | <0.0001 |
| **Heart failure** | 1.784 | 1.639–1.943 | <0.0001 |
| **Pulmonary embolism** | 1.517 | 1.299–1.772 | <0.0001 |
| **All-cause mortality** | 2.292 | 2.101–2.501 | <0.0001 |
| **Negative-control** | 0.921 | 0.668–1.271 | 0.6181 |

*The 2-year restricted follow-up sensitivity analysis used the same matched cohort framework as the main analysis, with follow-up censored at 730 days after index. Patients with the respective outcome before the time window were excluded from endpoint-specific Kaplan–Meier analyses. HRs are derived from platform-based Kaplan–Meier/hazard-ratio analyses; p values are from log-rank tests. MACE = major adverse cardiovascular events; HR = hazard ratio; CI = confidence interval; TT = testosterone therapy.*

**Supplementary Table S6. Outcomes for Age-stratified subgroup analyses (Sensitivity analyses)**

| *Endpoint* | *Age group* | *HR* | *95% CI* | *Log rank p* |
| --- | --- | --- | --- | --- |
| *MACE* | *18–54* | *1.384* | *1.275–1.501* | *<0.0001* |
|  | *55–75* | *1.467* | *1.409–1.527* | *<0.0001* |
| *Myocardial infarction* | *18–54* | *1.020* | *0.865–1.202* | *0.8168* |
|  | *55–75* | *1.042* | *0.969–1.122* | *0.2673* |
| *Ischaemic stroke* | *18–54* | *1.463* | *1.225–1.748* | *<0.0001* |
|  | *55–75* | *1.151* | *1.061–1.249* | *0.0007* |
| *All-cause mortality* | *18–54* | *1.460* | *1.312–1.625* | *<0.0001* |
|  | *55–75* | *1.915* | *1.815–2.021* | *<0.0001* |
| *Cardiac arrest* | *18–54* | *1.618* | *1.230–2.129* | *0.0005* |
|  | *55–75* | *1.279* | *1.097–1.492* | *0.0016* |
| *Pulmonary embolism* | *18–54* | *1.185* | *0.989–1.420* | *0.0659* |
|  | *55–75* | *1.079* | *0.979–1.189* | *0.1255* |
| *Heart failure* | *18–54* | *1.406* | *1.248–1.585* | *<0.0001* |
|  | *55–75* | *1.225* | *1.162–1.291* | *<0.0001* |
| *Negative control* | *18–54* | *1.115* | *0.896–1.389* | *0.3291* |
|  | *55–75* | *0.822* | *0.650–1.040* | *0.1021* |

*Exploratory age-stratified sensitivity analyses (18–54 and 55–75 years) within propensity-score–matched cohorts compared TT with versus without evidence of hypogonadism to assess robustness. Age strata were selected to better contrast younger and older patients. Associations were directionally consistent across strata with heterogeneity in magnitude: MACE risk was elevated in both younger and older men. Stroke and cardiac arrest signals were stronger in younger men, whereas excess all-cause mortality and heart-failure risk were more pronounced in older men; myocardial infarction and pulmonary embolism were not significant in either stratum. The primary outcome was MACE, defined as the composite of myocardial infarction, ischaemic stroke, cardiac arrest, and all-cause mortality. Abbreviations: CI, confidence interval; HR, hazard ratio; MACE, major adverse cardiovascular events; TT, testosterone therapy.*

**Supplementary Table S7. Outcomes for Race/ethnicity subgroup analyses**

| *Endpoint* | *Race/ethnicity* | *HR* | *95% CI* | *Log rank p* |
| --- | --- | --- | --- | --- |
| *MACE* | White | 1.494 | 1.434–1.556 | <0.0001 |
|  | Black or African American | 1.307 | 1.148–1.488 | <0.0001 |
|  | Asian | *2.391* | *1.779*–*3.214* | <0.0001 |
|  | Hispanic or Latino | 1.488 | 1.257–1.762 | <0.0001 |
| *Myocardial infarction* | White | 1.078 | 1.000–1.161 | 0.050 |
|  | Black or African American | 0.957 | 0.749–1.221 | 0.723 |
|  | Asian | *2.377* | *1.367*–*4.135* | 0.0016 |
|  | Hispanic or Latino | 0.884 | 0.638–1.223 | 0.456 |
| *Ischaemic stroke* | White | 1.256 | 1.155–1.366 | <0.0001 |
|  | Black or African American | 1.166 | 0.913–1.491 | 0.218 |
|  | Asian | *1.881* | *0.98*–3-61 | *0.0535* |
|  | Hispanic or Latino | 1.384 | 0.991–1.933 | 0.055 |
| *All-cause mortality* | White | 1.997 | 1.846–2.160 | <0.0001 |
|  | Black or African American | 1.505 | 1.267–1.787 | <0.0001 |
|  | Asian | *2.978* | *2.06*–4*.306* | <0.0001 |
|  | Hispanic or Latino | 1.815 | 1.445–2.279 | <0.0001 |
| *Cardiac arrest* | White | 1.528 | 1.309–1.784 | <0.0001 |
|  | Black or African American | 1.457 | 0.932–2.278 | 0.097 |
|  | Asian* | — | — | — |
|  | Hispanic or Latino | 2.459 | 1.330–4.548 | 0.003 |
| *Heart failure* | White | 1.581 | 1.481–1.688 | <0.0001 |
|  | Black or African American | 1.477 | 1.267–1.723 | <0.0001 |
|  | Asian | 2.106 | *1.316*–3.371 | *0.0015* |
|  | Hispanic or Latino | 1.295 | 1.021–1.641 | 0.033 |
| *Pulmonary embolism* | White | 1.307 | 1.192–1.433 | <0.0001 |
|  | Black or African American | 0.897 | 0.658–1.224 | 0.493 |
|  | Asian* | — | — | — |
|  | Hispanic or Latino | 1.115 | 0.727–1.710 | 0.618 |
| *Negative control* | White | 1.239 | 0.934–1.645 | 0.192 |
|  | Black or African American | 0.867 | 0.408–1.842 | 0.710 |
|  | Asian** | 0.719 | *0.413*–*1.253* | 0.2424 |
|  | Hispanic or Latino | 1.049 | 0.600–1.831 | 0.868 |

*Hazard ratios (HRs) and 95% confidence intervals (CIs) were estimated from the propensity-score–matched cohorts, comparing men receiving testosterone therapy (TT) with evidence of hypogonadism versus men receiving TT without evidence of hypogonadism within each race/ethnicity stratum. Race/ethnicity strata were defined using EHR-recorded categories as implemented in TriNetX. Log-rank p values reflect Kaplan–Meier comparisons within each stratum.*

** For selected endpoints in the Asian stratum, event counts were insufficient for stable time-to-event estimation; accordingly, HRs and log-rank p values are not reported.*

**** *As a negative-control outcome to probe residual confounding, acute appendicitis was used in the primary analyses. In the prespecified small Asian subgroup analysis, acute appendicitis occurred too infrequently to permit reliable estimation (event sparsity leading to non-estimable models). Therefore, for that subgroup analysis we revised the subgroup protocol and substituted 2 additional outcomes (acute appendicitis, burns or acute atopic conjunctivitis), a priori neutral negative-control outcome. This change was driven solely by event frequency considerations and is reported transparently.*

***Abbreviations:*** *CI, confidence interval; HR, hazard ratio; MACE, major adverse cardiovascular events; TT, testosterone therapy.*

**Supplementary Table S8 Outcomes in Asian subgroup after Matching**

| **Outcome** | **Case events** | **Control events** | **HR** | **95% CI** | **Log-rank p** |
| --- | --- | --- | --- | --- | --- |
| **MACE** | 148 | 63 | 2.391 | 1.779–3.214 | <0.0001 |
| **Myocardial infarction** | 42 | 18 | 2.377 | 1.367–4.135 | 0.0016 |
| **Ischaemic stroke** | 26 | 14 | 1.881 | 0.980–3.610 | 0.0535 |
| **All-cause mortality** | 112 | 38 | 2.978 | 2.060–4.306 | <0.0001 |
| **Heart failure overall** | 53 | 26 | 2.106 | 1.316–3.371 | 0.0015 |
| **Pulmonary embolism** | - | - | - | - | - |
| **Cardiac arrest** | - | - | - | - | - |

*Hazard ratios (HRs) were estimated using multivariable Cox proportional-hazards models in the full, unmatched cohorts, comparing TT users with evidence of hypogonadism versus TT users without evidence of hypogonadism (reference), with follow-up censored at 10 years. Models were adjusted for age at index; race/ethnicity (White, Black or African American, Asian, Hispanic or Latino); and baseline comorbidities/risk markers: chronic kidney disease, socioeconomic/psychosocial hazards, family history of ischaemic heart disease and other circulatory diseases, overweight/obesity, diabetes mellitus, essential hypertension, affective disorders, nicotine dependence, family history of diabetes mellitus, and neoplasms. MACE was defined as the composite of myocardial infarction, ischaemic stroke, or all-cause mortality. P values are two-sided. Pulmonary embolism and cardiac arrest results in the Asian subgroup were not displayed by the platform because patient counts were below the reporting threshold and were therefore not inferred. Case = TT without evidence of hypogonadism based on available structured EHR data; Control = TT with evidence of hypogonadism based on available structured EHR data*

**Abbreviations:** CI, confidence interval; HR, hazard ratio; MACE, major adverse cardiac and cerebrovascular events; TT, testosterone therapy.

**Supplementary Table S9. Multivariable Cox proportional-hazards models (full cohorts; 10 years follow up)**

| Endpoint | HR | 95% CI | p value |
| --- | --- | --- | --- |
| MACE | 1.438 | 1.395–1.482 | <0.0001 |
| All-cause mortality | 1.804 | 1.731–1.880 | <0.0001 |
| Myocardial infarction | 1.144 | 1.082–1.210 | <0.0001 |
| Ischaemic stroke | 1.164 | 1.093–1.240 | <0.0001 |
| Cardiac arrest | 1.559 | 1.390–1.749 | <0.0001 |
| Heart failure | 1.249 | 1.200–1.300 | <0.0001 |
| Pulmonary embolism | 1.036 | 0.964–1.114 | 0.3367 |

*Hazard ratios (HRs) were estimated using multivariable Cox proportional-hazards models in the full, unmatched cohorts, comparing TT users with evidence of hypogonadism versus TT users without evidence of hypogonadism (reference), with follow-up censored at 10 years. Models were adjusted for age at index; race/ethnicity (White, Black or African American, Asian, Hispanic or Latino); and baseline comorbidities/risk markers: chronic kidney disease, socioeconomic/psychosocial hazards, family history of ischaemic heart disease and other circulatory diseases, overweight/obesity, diabetes mellitus, essential hypertension, affective disorders, nicotine dependence, family history of diabetes mellitus, and neoplasms. MACE was defined as the composite of myocardial infarction, ischaemic stroke, or all-cause mortality. P values are two-sided.*

**Abbreviations:** CI, confidence interval; HR, hazard ratio; MACE, major adverse cardiac and cerebrovascular events; TT, testosterone therapy.

**Supplementary Table S10. Composition of exposure classification**

| Classification component | TT without evidence of hypogonadism | TT with evidence of hypogonadism |
| --- | --- | --- |
| ≥1 documented testosterone measurement before TT initiation, n (%) | 6,438 (6.7%) | — |
| Testosterone values above prespecified thresholds among those tested, n/N (%) | 6,438/6,438 (100%) | — |
| No documented testosterone measurement and no hypogonadism diagnostic code, n (%) | 89,228 (93.3%) | — |
| Diagnostic-code evidence present, regardless of laboratory evidence, n (%) | — | 164,206 (83.2%) |
| Laboratory evidence present, regardless of diagnostic-code evidence, n (%) | — | 136,300 (69.0%) |
| Both diagnostic and laboratory evidence, n (%) | — | 105,449 (53.4%) |

*Additional phenotyping variables were extracted in a contemporaneous descriptive TriNetX query. Because the TriNetX network is dynamic and saved-characteristic outputs depend on data availability and healthcare-organisation return at the time of extraction, the returned denominators for the expanded phenotyping query differed from the original pre-matching denominators.*

**Supplementary Table S11. Clinical outcomes in the laboratory-anchored sensitivity analysis**

| Outcome | Hazard ratio | Coefficient | Standard error | z | p value | 95% CI |
| --- | --- | --- | --- | --- | --- | --- |
| MACE | 1.519 | 0.418 | 0.061 | 6.860 | <0.0001 | 1.348–1.712 |
| All-cause mortality | 1.690 | 0.525 | 0.080 | 6.562 | <0.0001 | 1.445–1.976 |
| Ischaemic stroke | 1.430 | 0.358 | 0.133 | 2.697 | 0.0070 | 1.103–1.855 |
| Cardiac arrest | 1.665 | 0.510 | 0.225 | 2.261 | 0.0238 | 1.070–2.589 |
| Heart failure | 1.334 | 0.288 | 0.080 | 3.605 | 0.0003 | 1.140–1.560 |
| Pulmonary embolism | 1.096 | 0.092 | 0.162 | 0.566 | 0.5715 | 0.798–1.505 |

*This laboratory-anchored sensitivity analysis was restricted to patients with documented testosterone measurements before testosterone therapy initiation. The group without evidence of hypogonadism based on available structured EHR data was restricted to men with documented testosterone values above the prespecified biochemical thresholds and no evidence of hypogonadism or symptom-proxy codes before treatment initiation. The comparator group was restricted to men with at least two documented low testosterone measurements before treatment initiation, independent of diagnostic coding. Cox regression models were adjusted using the same covariate framework as the main analysis.*

**Abbreviations:** *MACE = major adverse cardiovascular events; CI = confidence interval; EHR = electronic health record.*

**Supplementary Table S12. Baseline characteristics before and after propensity-score matching – Subgroup age 18–54 years**

| **Characteristic** | **Before PSM** | | | **After PSM** | | |
| --- | --- | --- | --- | --- | --- | --- |
|  | **TT without hypogonadism**  **N=57,484** | **TT with hypogonadism N=98,524** | **SMD** | **TT without hypogonadism**  **N=54,877** | **TT with hypogonadism N=54,877** | **SMD** |
| **Age at index, years,** (mean ± SD) | *36.5 ± 10.4* | *37.4 ± 9.9* | *0.094* | *36.5 ± 10.4* | *36.5 ± 10.2* | *0.002* |
| **White,** n (%) | *42,229 (73.5%)* | *77,787 (79.0%)* | *0.129* | *42,229 (77.0%)* | *42,285 (77.1%)* | *0.002* |
| **Hispanic or Latino,** n (%) | *3,675 (6.4%)* | *7,504 (7.6%)* | *0.048* | *3,675 (6.7%)* | *3,620 (6.6%)* | *0.004* |
| **Black or African American,** n (%) | *3,150 (5.5%)* | *6,166 (6.3%)* | *0.033* | *3,150 (5.7%)* | *3,227 (5.9%)* | *0.006* |
| **Asian,** n (%) | *1,039 (1.8%)* | *2,074 (2.1%)* | *0.021* | *1,039 (1.9%)* | *923 (1.7%)* | *0.016* |
| **Chronic kidney disease,** n (%) | *509 (0.9%)* | *2,022 (2.1%)* | *0.097* | *501 (0.9%)* | *500 (0.9%)* | *<0.001* |
| **Socioeconomic/psychosocial hazards,** n (%) | *514 (0.9%)* | *2,510 (2.5%)* | *0.127* | *514 (0.9%)* | *529 (1.0%)* | *0.003* |
| **Family history of ischaemic heart disease,** n (%) | *618 (1.1%)* | *3,189 (3.2%)* | *0.149* | *618 (1.1%)* | *603 (1.1%)* | *0.003* |
| **Overweight and obesity,** n (%) | *4,010 (7.0%)* | *23,250 (23.6%)* | *0.475* | *4,010 (7.3%)* | *4,062 (7.4%)* | *0.004* |
| **Essential hypertension,** n (%) | *5,230 (9.1%)* | *24,706 (25.1%)* | *0.434* | *5,230 (9.5%)* | *5,182 (9.4%)* | *0.003* |
| **Affective disorders,** n (%) | *5,267 (9.2%)* | *20,518 (20.8%)* | *0.331* | *5,267 (9.6%)* | *5,221 (9.5%)* | *0.003* |
| **Nicotine dependence** n (%) | *2,883 (5.0%)* | *10,349 (10.5%)* | *0.206* | *2,883 (5.3%)* | *2,911 (5.3%)* | *0.002* |
| **Family history of diabetes mellitus,** n (%) | *386 (0.7%)* | *2,145 (2.2%)* | *0.127* | *386 (0.7%)* | *378 (0.7%)* | *0.002* |
| **Neoplasms,** n (%) | *3,944 (6.9%)* | *17,456 (17.7%)* | *0.335* | *3,944 (7.2%)* | *4,007 (7.3%)* | *0.004* |

*Baseline characteristics are shown before and after 1:1 propensity-score matching comparing TT with evidence of hypogonadism vs TT without evidence of hypogonadism, restricted to men aged 18–54 years at index. Interpretation of p values and SMD follows the same principles. Matching used greedy nearest-neighbour matching on the propensity score with a calliper of 0.1 SD of the logit. Covariate balance was assessed using standardised mean differences (SMD), with absolute SMD values closer to 0 indicating better balance. P values are descriptive (two-sided) and may be statistically significant even with minimal imbalance due to large sample size; SMD is emphasised for balance assessment.
Abbreviations: PSM, propensity-score matching; SMD, standardised mean difference; SD, standard deviation; TT, testosterone therapy; CKD, chronic kidney disease.*

**Supplementary Table S13. Baseline characteristics before and after propensity-score matching – Subgroup age 55–75 years**

| **Characteristic** | **Before PSM** | | | **After PSM** | | |
| --- | --- | --- | --- | --- | --- | --- |
|  | **TT without hypogonadism**  **N=** **78,332** | **TT with hypogonadism N=** **141,910** | **SMD** | **TT without hypogonadism**  **N=** **66,612** | **TT with hypogonadism N=** **66,612** | **SMD** |
| **Age at index, years,** (mean ± SD) | *58.1 ± 6.7* | *57.3 ± 6.9* | *0.125* | *57.5 ± 6.6* | *57.6 ± 6.6* | *0.015* |
| **White,** n (%) | *58,567 (74.8%)* | *114,168 (80.5%)* | *0.137* | *52,991 (79.6%)* | *52,683 (79.1%)* | *0.011* |
| **Hispanic or Latino,** n (%) | *3,055 (3.9%)* | *6,290 (4.4%)* | *0.027* | *2,672 (4.0%)* | *2,801 (4.2%)* | *0.010* |
| **Black or African American,** n (%) | *4,251 (5.4%)* | *11,685 (8.2%)* | *0.111* | *4,154 (6.2%)* | *4,107 (6.2%)* | *0.003* |
| **Asian,** n (%) | *1,063 (1.4%)* | *2,493 (1.8%)* | *0.032* | *1,037 (1.6%)* | *1,039 (1.6%)* | *<0.001* |
| **Chronic kidney disease,** n (%) | *1,515 (1.9%)* | *7,809 (5.5%)* | *0.189* | *1,477 (2.2%)* | *1,581 (2.4%)* | *0.010* |
| **Socioeconomic/psychosocial hazards,** n (%) | *302 (0.4%)* | *2,093 (1.5%)* | *0.114* | *300 (0.5%)* | *304 (0.5%)* | *0.001* |
| **Family history of ischaemic heart disease,** n (%) | *911 (1.2%)* | *5,315 (3.7%)* | *0.167* | *902 (1.4%)* | *924 (1.4%)* | *0.003* |
| **Overweight and obesity,** n (%) | *4,611 (5.9%)* | *31,361 (22.1%)* | *0.481* | *4,610 (6.9%)* | *4,700 (7.1%)* | *0.005* |
| **Essential hypertension,** n (%) | *12,757 (16.3%)* | *65,521 (46.2%)* | *0.681* | *12,752 (19.1%)* | *12,842 (19.3%)* | *0.003* |
| **Affective disorders,** n (%) | *4,318 (5.5%)* | *26,036 (18.3%)* | *0.404* | *4,308 (6.5%)* | *4,355 (6.5%)* | *0.003* |
| **Nicotine dependence** n (%) | *2,634 (3.4%)* | *12,963 (9.1%)* | *0.240* | *2,605 (3.9%)* | *2,712 (4.1%)* | *0.008* |
| **Family history of diabetes mellitus,** n (%) | *314 (0.4%)* | *2,344 (1.7%)* | *0.124* | *311 (0.5%)* | *332 (0.5%)* | *0.005* |
| **Neoplasms,** n (%) | *9,039 (11.5%)* | *40,499 (28.5%)* | *0.435* | *8,976 (13.5%)* | *9,250 (13.9%)* | *0.012* |

*Baseline characteristics are shown before and after 1:1 propensity-score matching comparing TT with evidence of hypogonadism vs TT without evidence of hypogonadism,* *restricted to men aged 55–75 years at index. Interpretation of p values and SMD follows the same principles. Matching used greedy nearest-neighbour matching on the propensity score with a calliper of 0.1 SD of the logit. Covariate balance was assessed using standardised mean differences (SMD), with absolute SMD values closer to 0 indicating better balance. P values are descriptive (two-sided) and may be statistically significant even with minimal imbalance due to large sample size; SMD is emphasised for balance assessment.
Abbreviations: PSM, propensity-score matching; SMD, standardised mean difference; SD, standard deviation; TT, testosterone therapy; CKD, chronic kidney disease.*

**Supplementary Table S14. Baseline characteristics before and after propensity-score matching – Subgroup Black or African American**

| **Characteristic** | **Before PSM** | | | **After PSM** | | |
| --- | --- | --- | --- | --- | --- | --- |
|  | **TT without hypogonadism**  **N=** **6,943** | **TT with hypogonadism N=** **17,472** | **SMD** | **TT without hypogonadism**  **N=** **6,906** | **TT with hypogonadism N=** **6,906** | **SMD** |
| **Age at index, years,** (mean ± SD) | *50.5 ± 11.5* | *51.1 ± 10.5* | *0.051* | *50.4 ± 11.5* | *50.6 ± 11.2* | *0.015* |
| **Chronic kidney disease,** n (%) | *265 (3.8%)* | *1,513 (8.7%)* | *0.201* | *265 (3.8%)* | *276 (4.0%)* | *0.008* |
| **Socioeconomic/psychosocial hazards,** n (%) | *43 (0.6%)* | *347 (2.0%)* | *0.121* | *43 (0.6%)* | *31 (0.4%)* | *0.024* |
| **Family history of ischaemic heart disease,** n (%) | *75 (1.1%)* | *596 (3.4%)* | *0.158* | *75 (1.1%)* | *57 (0.8%)* | *0.027* |
| **Overweight and obesity,** n (%) | *604 (8.7%)* | *5,135 (29.4%)* | *0.546* | *604 (8.7%)* | *617 (8.9%)* | *0.007* |
| **Essential hypertension,** n (%) | *1,362 (19.6%)* | *8,793 (50.3%)* | *0.680* | *1,362 (19.7%)* | *1,368 (19.8%)* | *0.002* |
| **Affective disorders,** n (%) | *394 (5.7%)* | *2,460 (14.1%)* | *0.285* | *394 (5.7%)* | *368 (5.3%)* | *0.016* |
| **Nicotine dependence** n (%) | *379 (5.5%)* | *2,044 (11.7%)* | *0.224* | *379 (5.5%)* | *390 (5.6%)* | *0.007* |
| **Family history of diabetes mellitus,** n (%) | *48 (0.7%)* | *457 (2.6%)* | *0.151* | *48 (0.7%)* | *39 (0.6%)* | *0.016* |
| **Neoplasms,** n (%) | *568 (8.2%)* | *4,140 (23.7%)* | *0.434* | *568 (8.2%)* | *584 (8.5%)* | *0.008* |

*Baseline characteristics are presented before and after 1:1 propensity-score matching, comparing testosterone therapy (TT) in men with evidence of hypogonadism versus TT in men without evidence of hypogonadism, restricted to Black or African American men. Interpretation of p values and SMD follows the same principles. Matching used greedy nearest-neighbour matching on the propensity score with a calliper of 0.1 SD of the logit. Covariate balance was assessed using standardised mean differences (SMD), with absolute SMD values closer to 0 indicating better balance. P values are descriptive (two-sided) and may be statistically significant even with minimal imbalance due to large sample size; SMD is emphasised for balance assessment.
Abbreviations: PSM, propensity-score matching; SMD, standardised mean difference; SD, standard deviation; TT, testosterone therapy; CKD, chronic kidney disease.*

**Supplementary Table S15. Baseline characteristics before and after propensity-score matching – Subgroup White**

| **Characteristic** | **Before PSM** | | | **After PSM** | | |
| --- | --- | --- | --- | --- | --- | --- |
|  | **TT without hypogonadism**  **N=** **94,682** | **TT with hypogonadism N=** **185,418** | **SMD** | **TT without hypogonadism**  **N=** **88,652** | **TT with hypogonadism N=** **88,652** | **SMD** |
| **Age at index, years,** (mean ± SD) | *51.2 ± 11.4* | *50.7 ± 11.1* | *0.048* | *50.4 ± 11.2* | *50.4 ± 11.1* | *0.002* |
| **Chronic kidney disease,** n (%) | *1,352 (1.4%)* | *6,972 (3.8%)* | *0.147* | *1,349 (1.5%)* | *1,362 (1.5%)* | *0.001* |
| **Socioeconomic/psychosocial hazards,** n (%) | *523 (0.6%)* | *3,329 (1.8%)* | *0.116* | *523 (0.6%)* | *506 (0.6%)* | *0.003* |
| **Family history of ischaemic heart disease,** n (%) | *1,234 (1.3%)* | *7,115 (3.8%)* | *0.161* | *1,233 (1.4%)* | *1,144 (1.3%)* | *0.009* |
| **Overweight and obesity,** n (%) | *6,512 (6.9%)* | *42,837 (23.1%)* | *0.467* | *6,512 (7.3%)* | *6,590 (7.4%)* | *0.003* |
| **Essential hypertension,** n (%) | *14,025 (14.8%)* | *72,424 (39.1%)* | *0.568* | *14,025 (15.8%)* | *14,024 (15.8%)* | *<0.001* |
| **Affective disorders,** n (%) | *7,036 (7.4%)* | *38,188 (20.6%)* | *0.386* | *7,036 (7.9%)* | *7,021 (7.9%)* | *0.001* |
| **Nicotine dependence** n (%) | *4,378 (4.6%)* | *19,192 (10.4%)* | *0.219* | *4,377 (4.9%)* | *4,420 (5.0%)* | *0.002* |
| **Family history of diabetes mellitus,** n (%) | *445 (0.5%)* | *3,242 (1.7%)* | *0.122* | *445 (0.5%)* | *425 (0.5%)* | *0.003* |
| **Neoplasms,** n (%) | *9,759 (10.3%)* | *46,166 (24.9%)* | *0.390* | *9,759 (11.0%)* | *9,786 (11.0%)* | *0.001* |

*Baseline characteristics are presented before and after 1:1 propensity-score matching, comparing testosterone therapy (TT) in men with evidence of hypogonadism versus TT in men without evidence of hypogonadism, restricted to White men. Interpretation of p values and SMD follows the same principles. Matching used greedy nearest-neighbour matching on the propensity score with a calliper of 0.1 SD of the logit. Covariate balance was assessed using standardised mean differences (SMD), with absolute SMD values closer to 0 indicating better balance. P values are descriptive (two-sided) and may be statistically significant even with minimal imbalance due to large sample size; SMD is emphasised for balance assessment.
Abbreviations: PSM, propensity-score matching; SMD, standardised mean difference; SD, standard deviation; TT, testosterone therapy; CKD, chronic kidney disease.*

**Supplementary Table S16. Baseline characteristics before and after propensity-score matching – Subgroup Asian**

| **Characteristic** | **Before PSM** | | | **After PSM** | | |
| --- | --- | --- | --- | --- | --- | --- |
|  | **TT without hypogonadism**  **N=** **1,650** | **TT with hypogonadism N=** **4,084** | **SMD** | **TT without hypogonadism**  **N=** **1,639** | **TT with hypogonadism N=** **1,639** | **SMD** |
| **Age at index, years,** (mean ± SD) | *47.1 ± 12.6* | *49.8 ± 11.4* | *0.2292* | *47.3 ± 12.4* | *47.4 ± 11.9* | *0.0123* |
| **Chronic kidney disease,** n (%) | *45 (2.7%)* | *284 (6.9%)* | *0.1979* | *45 (2.7%)* | *51 (3.1%)* | *0.0217* |
| **Socioeconomic/psychosocial hazards,** n (%) | *10 (0.6%)* | *64 (1.6%)* | *0.0928* | *10 (0.6%)* | *10 (0.6%)* | *<0.0001* |
| **Family history of ischaemic heart disease,** n (%) | *18 (1.1%)* | *132 (3.2%)* | *0.1476* | *18 (1.098%)* | *15 (0.9%)* | *0.0183* |
| **Overweight and obesity,** n (%) | *70 (4.2%)* | *706 (17.3%)* | *0.4305* | *70 (4.3%)* | *68 (4.1%)* | *0.0061* |
| **Essential hypertension,** n (%) | *210 (12.7%)* | *1,520 (37.2%)* | *0.5899* | *210 (12.8%)* | *203 (12.4%)* | *0.0129* |
| **Affective disorders,** n (%) | *61 (3.7%)* | *438 (10.7%)* | *0.2742* | *61 (3.7%)* | *59 (3.6%)* | *0.0065* |
| **Nicotine dependence** n (%) | *39 (2.3%)* | *265 (6.5%)* | *0.2016* | *39 (2.4%)* | *36 (2.2%)* | *0.0122* |
| **Family history of diabetes mellitus,** n (%) | *10 (0.6%)* | *91 (2.2%)* | *0.1376* | *10 (0.6%)* | *10 (0.6%)* | *<0.0001* |
| **Neoplasms,** n (%) | *161 (9.8%)* | *1,187 (29.0%)* | *0.5034* | *161 (9.8%)* | *173 (10.5%)* | *0.0242* |

*Baseline characteristics are presented before and after 1:1 propensity-score matching, comparing testosterone therapy (TT) in men with evidence of hypogonadism versus TT in men without evidence of hypogonadism, restricted to men of Asian race. Interpretation of p values and SMD follows the same principles. Matching used greedy nearest-neighbour matching on the propensity score with a calliper of 0.1 SD of the logit. Covariate balance was assessed using standardised mean differences (SMD), with absolute SMD values closer to 0 indicating better balance. P values are descriptive (two-sided) and may be statistically significant even with minimal imbalance due to large sample size; SMD is emphasised for balance assessment.
Abbreviations: PSM, propensity-score matching; SMD, standardised mean difference; SD, standard deviation; TT, testosterone therapy; CKD, chronic kidney disease.*

**Supplementary Table S17. Baseline characteristics before and after propensity-score matching – Subgroup Hispanic or Latino**

| **Characteristic** | **Before PSM** | | | **After PSM** | | |
| --- | --- | --- | --- | --- | --- | --- |
|  | **TT without hypogonadism**  **N=** **6,077** | **TT with hypogonadism N=** **12,764** | **SMD** | **TT without hypogonadism**  **N=** **5,789** | **TT with hypogonadism N=** **5,789** | **SMD** |
| **Age at index, years,** (mean ± SD) | *48.0 ± 11.5* | *47.8 ± 11.0* | *0.020* | *47.3 ± 11.3* | *47.2 ± 11.1* | *0.006* |
| **Chronic kidney disease,** n (%) | *136 (2.2%)* | *537 (4.2%)* | *0.112* | *131 (2.3%)* | *111 (1.9%)* | *0.024* |
| **Socioeconomic/psychosocial hazards,** n (%) | *42 (0.7%)* | *304 (2.4%)* | *0.138* | *42 (0.7%)* | *30 (0.5%)* | *0.026* |
| **Family history of ischaemic heart disease,** n (%) | *76 (1.3%)* | *461 (3.6%)* | *0.154* | *76 (1.3%)* | *58 (1.0%)* | *0.029* |
| **Overweight and obesity,** n (%) | *580 (9.5%)* | *3,645 (28.6%)* | *0.499* | *580 (10.0%)* | *604 (10.4%)* | *0.014* |
| **Essential hypertension,** n (%) | *930 (15.3%)* | *4,711 (36.9%)* | *0.507* | *930 (16.1%)* | *908 (15.7%)* | *0.010* |
| **Affective disorders,** n (%) | *482 (7.9%)* | *2,510 (19.7%)* | *0.345* | *482 (8.3%)* | *493 (8.5%)* | *0.007* |
| **Nicotine dependence** n (%) | *288 (4.7%)* | *1,298 (10.2%)* | *0.208* | *288 (5.0%)* | *290 (5.0%)* | *0.002* |
| **Family history of diabetes mellitus,** n (%) | *50 (0.8%)* | *465 (3.6%)* | *0.192* | *50 (0.9%)* | *36 (0.6%)* | *0.028* |
| **Neoplasms,** n (%) | *619 (10.2%)* | *3,051 (23.9%)* | *0.371* | *619 (10.7%)* | *630 (10.9%)* | *0.006* |

*Baseline characteristics are presented before and after 1:1 propensity-score matching, comparing testosterone therapy (TT) in men with evidence of hypogonadism versus TT in men without evidence of hypogonadism, restricted to those of Hispanic or Latino ethnicity. Interpretation of p values and SMD follows the same principles. Matching used greedy nearest-neighbour matching on the propensity score with a calliper of 0.1 SD of the logit. Covariate balance was assessed using standardised mean differences (SMD), with absolute SMD values closer to 0 indicating better balance. P values are descriptive (two-sided) and may be statistically significant even with minimal imbalance due to large sample size; SMD is emphasised for balance assessment.
Abbreviations: PSM, propensity-score matching; SMD, standardised mean difference; SD, standard deviation; TT, testosterone therapy; CKD, chronic kidney disease.*

**Supplementary Table S18. Baseline characteristics before and after propensity-score matching – Subgroup excluding patients with diabetes mellitus**

| **Characteristic** | **Before PSM** | | | **After PSM** | | |
| --- | --- | --- | --- | --- | --- | --- |
|  | **TT without hypogonadism**  **N=** **106,620** | **TT with hypogonadism N=** **136,827** | **SMD** | **TT without hypogonadism**  **N=** **85,290** | **TT with hypogonadism N=** **85,290** | **SMD** |
| **Age at index, years,** (mean ± SD) | *51.1 ± 11.5* | *48.8 ± 11.4* | *0.198* | *49.4 ± 11.6* | *49.4 ± 11.4* | *0.002* |
| **Chronic kidney disease,** n (%) | *1,624 (1.5%)* | *3,287 (2.4%)* | *0.063* | *1,468 (1.7%)* | *1,388 (1.6%)* | *0.007* |
| **Socioeconomic/psychosocial hazards,** n (%) | *649 (0.6%)* | *2,619 (1.9%)* | *0.117* | *643 (0.8%)* | *660 (0.8%)* | *0.002* |
| **Family history of ischaemic heart disease,** n (%) | *1,412 (1.3%)* | *4,678 (3.4%)* | *0.138* | *1,388 (1.6%)* | *1,381 (1.6%)* | *0.001* |
| **Overweight and obesity,** n (%) | *6,436 (6.0%)* | *24,109 (17.6%)* | *0.365* | *6,414 (7.5%)* | *6,492 (7.6%)* | *0.003* |
| **Essential hypertension,** n (%) | *14,415 (13.5%)* | *41,086 (30.0%)* | *0.408* | *14,278 (16.7%)* | *14,141 (16.6%)* | *0.004* |
| **Affective disorders,** n (%) | *6,675 (6.3%)* | *24,189 (17.7%)* | *0.357* | *6,648 (7.8%)* | *6,791 (8.0%)* | *0.006* |
| **Nicotine dependence** n (%) | *4,144 (3.9%)* | *12,531 (9.2%)* | *0.215* | *4,082 (4.8%)* | *4,283 (5.0%)* | *0.011* |
| **Family history of diabetes mellitus,** n (%) | *549 (0.5%)* | *1,974 (1.4%)* | *0.094* | *543 (0.6%)* | *502 (0.6%)* | *0.006* |
| **Neoplasms,** n (%) | *10,320 (9.7%)* | *31,129 (22.8%)* | *0.360* | *10,229 (12.0%)* | *10,587 (12.4%)* | *0.013* |

*Baseline characteristics are shown before and after 1:1 propensity-score matching comparing TT with evidence of hypogonadism vs TT without evidence of hypogonadism,* *restricted to men excluding patients with diabetes mellitus at index. Interpretation of p values and SMD follows the same principles. Matching used greedy nearest-neighbour matching on the propensity score with a calliper of 0.1 SD of the logit. Covariate balance was assessed using standardised mean differences (SMD), with absolute SMD values closer to 0 indicating better balance. P values are descriptive (two-sided) and may be statistically significant even with minimal imbalance due to large sample size; SMD is emphasised for balance assessment.
Abbreviations: PSM, propensity-score matching; SMD, standardised mean difference; SD, standard deviation; TT, testosterone therapy; CKD, chronic kidney disease.*

**Supplementary Table S19. Baseline characteristics before and after propensity-score matching stratified by haematocrit level in men receiving testosterone therapy without of hypogonadism**

| **Characteristic** | **Before PSM** | | | **After PSM** | | |
| --- | --- | --- | --- | --- | --- | --- |
|  | **TT without hypogonadism - Hct >50%**  **N=** ***10,156*** | **TT without hypogonadism - Hct 41–50%**  **N=** ***50,222*** | **SMD** | **TT without hypogonadism - Hct >50%**  **N=** ***10,154*** | **TT without hypogonadism - Hct 41–50%**  **N=** ***10,154*** | **SMD** |
| **Age at index, years,** (mean ± SD) | *52.0 ± 11.1* | *51.9 ± 11.6* | *0.012* | *52.0 ± 11.1* | *51.9 ± 11.1* | *0.010* |
| **Chronic kidney disease,** n (%) | *567 (5.6%)* | *2,109 (4.2%)* | *0.064* | *565 (5.6%)* | *523 (5.2%)* | *0.018* |
| **Socioeconomic/psychosocial hazards,** n (%) | *221 (2.2%)* | *783 (1.6%)* | *0.046* | *219 (2.2%)* | *210 (2.1%)* | *0.006* |
| **Family history of ischaemic heart disease,** n (%) | *495 (4.9%)* | *1,798 (3.6%)* | *0.064* | *493 (4.9%)* | *476 (4.7%)* | *0.008* |
| **Overweight and obesity,** n (%) | *2,012 (19.8%)* | *7,752 (15.4%)* | *0.115* | *2,010 (19.8%)* | *2,009 (19.8%)* | *<0.001* |
| **Essential hypertension,** n (%) | *3,711 (36.5%)* | *15,832 (31.5%)* | *0.106* | *3,709 (36.5%)* | *3,711 (36.5%)* | *<0.001* |
| **Affective disorders,** n (%) | *1,588 (15.6%)* | *6,804 (13.5%)* | *0.059* | *1,587 (15.6%)* | *1,566 (15.4%)* | *0.006* |
| **Nicotine dependence** n (%) | *1,190 (11.7%)* | *4,473 (8.9%)* | *0.092* | *1,188 (11.7%)* | *1,185 (11.7%)* | *0.001* |
| **Family history of diabetes mellitus,** n (%) | *176 (1.7%)* | *690 (1.4%)* | *0.029* | *176 (1.7%)* | *148 (1.5%)* | *0.022* |
| **Neoplasms,** n (%) | *1,896 (18.7%)* | *9,137 (18.2%)* | *0.012* | *1,895 (18.7%)* | *1,827 (18.0%)* | *0.017* |

*This sensitivity analysis was restricted to men receiving testosterone therapy (TT) without evidence of hypogonadism and aimed to probe whether baseline haematocrit (Hct)—a clinically relevant safety marker under TT—was associated with differential baseline risk profiles after balancing for measured confounders. Patients were stratified by baseline haematocrit into Hct >50% versus Hct 41–50%. The index date was defined as the first qualifying TT record in accordance with the primary study definition. Baseline covariates were ascertained within the prespecified pre-index window used in the main analysis. To mitigate confounding, we performed 1:1 propensity-score matching (greedy nearest-neighbour matching; calliper 0.1 SD of the logit of the propensity score) using the same covariate set as in the primary analysis. Balance was quantified using standardised mean differences (SMDs), with values <0.10 generally indicating acceptable balance; in this table, post-match balance was excellent overall (typically SMD <0.05). Continuous variables are shown as mean ± SD and categorical variables as n (%).*

*Abbreviations: Hct, haematocrit; PSM, propensity-score matching; SD, standard deviation; SMD, standardised mean difference; TT, testosterone therapy.*

**Supplementary Table S20. Outcomes in the sensitivity analysis excluding patients with diabetes mellitus**

| *Endpoint* | *HR* | *95% CI* | *Log rank p* |
| --- | --- | --- | --- |
| *MACE* | *1.765* | *1.686–1.847* | *<0.001* |
| *Myocardial infarction* | *1.594* | *1.459–1.740* | *<0.001* |
| *Ischaemic stroke* | *1.607* | *1.464–1.765* | *<0.001* |
| *All-cause mortality* | *1.953* | *1.840–2.073* | *<0.001* |
| *Cardiac arrest* | *1.804* | *1.519–2.143* | *<0.001* |
| *Pulmonary embolism* | *1.322* | *1.185–1.475* | *<0.001* |
| *Heart failure* | *1.933* | *1.814–2.059* | *<0.001* |
| *Negative control* | *0.845* | *0.687–1.039* | *0.109* |

*This sensitivity analysis excluded patients with diabetes mellitus at baseline and repeated the primary design within propensity-score–matched cohorts comparing testosterone therapy (TT) without evidence of hypogonadism versus TT with evidence of hypogonadism. MACE was defined as the composite of myocardial infarction, ischaemic stroke, cardiac arrest, heart failure, and all-cause mortality. The negative-control endpoint was acute appendicitis.*

**Abbreviations:** CI, confidence interval; HR, hazard ratio; MACE, major adverse cardiovascular events; TT, testosterone therapy.

**Supplementary Table S21. Haematocrit-stratified outcomes among men receiving testosterone therapy without evidence of hypogonadism**

| *Endpoint* | *HR* | *95% CI* | *Log rank p* |
| --- | --- | --- | --- |
| *MACE* | *1.120* | *1.002–1.251* | *0.046* |
| *Myocardial infarction* | *1.267* | *1.046–1.536* | *0.015* |
| *Ischaemic stroke* | *1.118* | *0.884–1.413* | *0.351* |
| *All-cause mortality* | *1.042* | *0.911–1.193* | *0.550* |
| *Cardiac arrest* | *1.416* | *0.982–2.041* | *0.061* |
| *Pulmonary embolism* | *1.123* | *0.863–1.461* | *0.388* |
| *Heart failure* | *1.057* | *0.918–1.217* | *0.443* |
| *Negative control* | *0.765* | *0.394–1.485* | *0.427* |

*This prespecified sensitivity analysis was restricted to men receiving testosterone therapy (TT) without evidence of hypogonadism and examined whether baseline haematocrit (Hct), a key safety marker during TT, was associated with differential long-term outcomes. Patients were stratified by baseline Hct >50% versus Hct 41–50% and compared using 1:1 propensity-score matching (greedy nearest-neighbour; calliper 0.1 SD of the logit). Index definition, outcome ascertainment, follow-up, and censoring were identical to the primary analysis. MACE comprised myocardial infarction, ischaemic stroke, cardiac arrest, heart failure, and all-cause mortality.*

*Abbreviations: CI, confidence interval; Hct, haematocrit; HR, hazard ratio; MACE, major adverse cardiovascular events; TT, testosterone therapy.*

**Supplementary Table S22. Heterogeneity testing across race-specific matched estimates**

| Outcome | White HR (95% CI) | Black/African American HR (95% CI) | Asian HR (95% CI) | p for heterogeneity |
| --- | --- | --- | --- | --- |
| MACE | 1.494 (1.434–1.556) | 1.307 (1.148–1.488) | 2.391 (1.779–3.214) | 0.0010 |
| Myocardial infarction | 1.078 (1.000–1.161) | 0.957 (0.749–1.221) | 2.377 (1.367–4.135) | 0.0126 |
| Ischaemic stroke | 1.256 (1.155–1.366) | 1.166 (0.913–1.491) | 1.881 (0.980–3.610) | 0.4014 |
| All-cause mortality | 1.883 (1.782–1.990) | 1.505 (1.267–1.787) | 2.978 (2.060–4.306) | 0.0022 |
| Heart failure overall | 1.289 (1.221–1.360) | 1.477 (1.267–1.723) | 2.106 (1.316–3.371) | 0.0374 |

*Race-specific estimates were derived from matched race-stratified Kaplan–Meier/Cox analyses. Heterogeneity p values were calculated from race-specific log hazard ratios and standard errors derived from the corresponding 95% confidence intervals. Pulmonary embolism and cardiac arrest were not included because Asian subgroup estimates were not displayed due to small cell counts.*

**Supplementary Table S23. Extended Baseline characteristics**

| Characteristic | TT without documented evidence of hypogonadism | TT with documented evidence of hypogonadism |
| --- | --- | --- |
| Nutritional anaemias, n (%) | 947 (1.0%) | 6,697 (3.5%) |
| Haemolytic anaemias, n (%) | 206 (0.2%) | 1,720 (0.9%) |
| Aplastic/other anaemias and bone marrow failure, n (%) | 2,584 (2.7%) | 14,782 (7.8%) |
| Secondary polycythaemia, n (%) | 553 (0.6%) | 4,817 (2.6%) |
| Pituitary disorders, n (%) | 1,383 (1.5%) | 14,484 (7.7%) |
| Hyperprolactinaemia, n (%) | 92 (0.1%) | 1,999 (1.1%) |
| Delayed puberty, n (%) | 96 (0.1%) | 440 (0.2%) |
| Male infertility, n (%) | 197 (0.2%) | 3,689 (2.0%) |
| Acquired absence of genital organ(s), n (%) | 195 (0.2%) | 2,004 (1.1%) |
| Malignant neoplasm of testis, n (%) | 320 (0.3%) | 2,163 (1.1%) |
| Personal history of malignant neoplasm of testis, n (%) | 205 (0.2%) | 1,417 (0.8%) |
| Simple orchiectomy, n (%) | 16 (0.0%) | 254 (0.1%) |
| Radical orchiectomy for tumour, n (%) | 23 (0.0%) | 584 (0.3%) |
| Orchiopexy, n (%) | 20 (0.0%) | 85 (0.0%) |

*Additional phenotyping variables were extracted in a contemporaneous descriptive TriNetX query. Because the TriNetX network is dynamic and saved-characteristic outputs depend on data availability and healthcare-organisation return at the time of extraction, the returned denominators for the expanded phenotyping query differed from the original pre-matching denominators. The returned denominators for this supplementary query were 95,144 for TT without documented evidence of hypogonadism and 188,029 for TT with documented evidence of hypogonadism. No imputation was performed.*

**Supplementary Table S24. Availability of key baseline measurements before testosterone therapy initiation**

| Measurement | TT without evidence of hypogonadism | Availability | TT with evidence of hypogonadism | Availability |
| --- | --- | --- | --- | --- |
| BMI, kg/m² | 31.5 ± 6.6 | 49% | 32.5 ± 6.9 | 74% |
| Haematocrit, % | 42.9 ± 6.8 | 33% | 43.4 ± 7.3 | 74% |
| Haemoglobin, g/dL | 14.5 ± 2.1 | 32% | 14.8 ± 1.7 | 72% |
| Erythrocytes | 4.83 ± 0.79 | 32% | 4.88 ± 0.81 | 71% |
| Leukocytes | 9.96 ± 89.6 | 30% | 12.0 ± 144 | 68% |
| Creatinine, mg/dL | 1.14 ± 2.64 | 33% | 1.31 ± 6.22 | 73% |
| eGFR, mL/min/1.73 m² | 84.0 ± 26.7 | 33% | 85.6 ± 23.8 | 73% |
| Urea nitrogen, mg/dL | 16.5 ± 8.6 | 31% | 16.2 ± 6.3 | 70% |
| Sodium, mmol/L | 139 ± 2.9 | 32% | 139 ± 2.6 | 72% |
| Potassium, mmol/L | 4.19 ± 0.45 | 32% | 4.27 ± 0.41 | 73% |
| Chloride, mmol/L | 103 ± 4.4 | 32% | 103 ± 3.8 | 72% |
| Bicarbonate, mmol/L | 26.3 ± 3.3 | 31% | 26.3 ± 3.0 | 71% |

*Availability indicates the proportion of patients with a structured measurement available before testosterone therapy initiation. Missing laboratory or anthropometric values were not imputed. Diagnosis-, procedure-, and medication-based covariates were handled separately as recorded structured EHR variables; absence of a recorded code was interpreted as absence of recorded evidence in the available structured data, not as definitive clinical absence.*
